# Analysis of Immune Cell Infiltration Distribution and Prognostic Value in Obstructive Colorectal Cancer

**DOI:** 10.3390/biomedicines13112596

**Published:** 2025-10-23

**Authors:** Yifan Xue, Zhenxing Jiang, Junnan Gu, Shenghe Deng, Kailin Cai, Ke Wu

**Affiliations:** Department of Gastrointestinal Surgery, Union Hospital, Tongji Medical College, Huazhong University of Science and Technology, 1277 Jiefang Rd, Wuhan 430022, China; xueyifan_dsa@163.com (Y.X.); d202482252@hust.edu.cn (Z.J.); gujunnan@hust.edu.cn (J.G.); dengshenghe@hust.edu.cn (S.D.)

**Keywords:** colorectal cancer, intestinal obstruction, tumor immune microenvironment, tumor-infiltrating immune cells, immune spatial redistribution

## Abstract

**Objective**: This study aims to determine how intestinal obstruction influences the tumor immune microenvironment (TIME) and its impact on prognosis in colorectal cancer (CRC). **Methods**: Immune cell densities (CD4^+^, CD8^+^, CD20^+^, CD68^+^) within central tumor (CT) and invasive margin (IM) compartments were quantitatively analyzed using immunohistochemistry (IHC) and QuPath digital pathology in surgical resection samples from 328 patients (164 obstructed colon cancer [OCRC] vs. 164 non-obstructed [NOCRC], cohorts matched by propensity scoring). Findings on tumor-infiltrating immune cell spatial distribution were integrated with peripheral blood immune cell counts and clinicopathological characteristics to characterize the obstructed colon cancer immune microenvironment. Associations with disease-free survival (DFS) and overall survival (OS) were evaluated. **Results**: OCRC exhibited higher lymphocytic infiltration, particularly in the CT compartment, compared to NOCRC, with significantly elevated CT-CD8^+^ T cell density in T4-stage OCRC (*p* < 0.005). Obstruction enhanced immune cell correlations across compartments, especially in T4 tumors, and the CD68^+^/CD8^+^ ratio effectively discriminated obstruction status (CT area under the curve (AUC): T4 = 0.879). Peripheral lymphocytopenia was pronounced in obstructive cases (*p* = 0.003). Critically, T4 OCRC showed a complete loss of all correlations between tumor-infiltrating immune cells and peripheral parameters. Despite increased infiltration, high CD8^+^ density in OCRC correlated with worse prognosis, indicating a paradoxical role influenced by obstruction context. CD68^+^ macrophages in the invasive margin consistently predicted improved survival (Disease-free survival [DFS]: Hazard ratio [HR] = 0.59, *p* = 0.008). **Conclusions**: Intestinal obstruction in CRC, particularly in T4-stage tumors, may represent an immunologically active state that alters local immune infiltration and systemic–local immune crosstalk. These findings suggest that obstruction status could refine prognostic stratification and inform therapeutic strategies, although validation in larger cohorts is warranted.

## 1. Introduction

Colorectal cancer (CRC) persists as a leading cause of global cancer mortality, representing the third most prevalent malignancy worldwide [1]. Despite improvements in screening programs and therapeutic strategies, a substantial proportion of patients continue to present with advanced-stage disease in China [2]. Among the serious complications of CRC, intestinal obstruction poses significant clinical challenges. Occurring in approximately 25–40% of CRC patients, obstruction arises when tumor masses or adhesions impede intestinal content flow, leading to nausea, vomiting, abdominal pain, and dehydration that often necessitate urgent hospitalization. While untreated obstruction can be fatal, its acute presentation may serve as the initial manifestation of CRC, prompting surgical intervention. Surgeons universally recognize the therapeutic complexity and poor prognosis associated with malignant bowel obstruction, making it a frequent indication for palliative surgical consultation [3].

Beyond its clinical urgency, intestinal obstruction fundamentally alters the tumor microenvironment. Research indicates that obstructed CRC (OCRC) exhibits distinct extracellular matrix (ECM) remodeling characterized by increased collagen deposition, proteoglycan alterations, and biomechanical stiffening. These changes correlate with the accumulation of palmitic acid (PA), which activates the NF-κB pathway in tumor cells to stimulate cytokine secretion (e.g., CSF1, TGFβ1, CXCL8). This cascade promotes the activation of matrix cancer-associated fibroblasts (mCAFs) that exacerbate ECM stiffening—a hallmark of obstruction pathophysiology [4,5]. Critically, this mechanically altered microenvironment coincides with immune reprogramming, though the specific immunological shifts remain poorly characterized.

The tumor immune microenvironment (TIME) constitutes a pivotal determinant of CRC progression and therapeutic response. Its composition—defined by the type, density, functional orientation, and spatial distribution of infiltrating immune cells—holds significant prognostic value. High densities of CD3^+^ and CD8^+^ T cells within both the central tumor (CT) and invasive margin (IM) correlate with improved survival, forming the basis of the Consensus Immunoscore that outperforms traditional Tumor, Node, Metastasis (TNM) staging system in prognostic accuracy [6]. However, emerging evidence highlights the contextual plasticity of immune infiltrates. For instance, while CD8^+^ T cells typically confer protection, their functional state can shift toward exhaustion under specific microenvironmental conditions such as chronic inflammation or metabolic stress. Similarly, macrophages exhibit dual roles: M1-like phenotypes exert antitumor activity at tumor-host interfaces, whereas M2-like polarization promotes immunosuppression and angiogenesis [7]. These nuances underscore that immune contexture must be interpreted within specific pathological and anatomical constraints.

Mounting evidence suggests that mechanical stress and gut barrier disruption in obstruction may critically reshape immune responses. The gut microbiota, a key modulator of intestinal immunity, demonstrates spatial heterogeneity across tumor regions. Obstruction-induced barrier breakdown likely exacerbates microbial translocation, potentially driving inflammation through Toll-like receptor activation and neutrophil recruitment. Additionally, the Consensus Molecular Subtype (CMS) classification reveals that CMS4 tumors—characterized by TGF-β activation, stromal infiltration, and epithelial–mesenchymal transition—are overrepresented in obstruction contexts [8,9]. This subtype exhibits distinct immune evasion mechanisms, including T-cell exclusion and myeloid cell skewing. Despite these insights, no controlled studies have systematically compared TIME profiles between obstructed and non-obstructed CRC (NOCRC). The high incidence of CRC-associated obstruction and its established biological severity underscore an urgent need to investigate its immunological consequences [10].

## 2. Materials and Methods

### 2.1. Observational Methods

#### 2.1.1. Clinical Data

This study included patients with pathologically confirmed primary colon cancer who underwent complete (R0) surgical resection at the Department of Gastrointestinal Surgery, Union Hospital, Tongji Medical College, Huazhong University of Science and Technology (Wuhan, China) between 1 January 2013, and 1 September 2021. Patients transferred from other medical institutions were also eligible. Patients presenting with preoperative intestinal obstruction were assigned to the obstruction group (experimental group), while patients without obstruction comprised the control group. The Institutional Review Board of Union Hospital, Tongji Medical College, Huazhong University of Science and Technology approved all procedures (No.2018-S377). The study was conducted in accordance with the ethical principles of the Declaration of Helsinki.

Inclusion Criteria: (1) Primary diagnosis of colorectal cancer. (2) For the experimental group: Documented diagnosis of intestinal obstruction occurring at the time of or subsequent to the initial colon cancer diagnosis but prior to surgical intervention. Absence of any prior history of intestinal obstruction. (3) Absence of concurrent or prior diagnoses of other malignancies that could significantly impact overall survival assessment. (4) Complete surgical resection (R0) of the primary tumor. Exclusion Criteria: (1) History of any cancer-related surgery or abdominal surgery occurring between the initial colon cancer diagnosis and the documented obstruction event (for the experimental group). (2) Incomplete tumor resection (non-R0 resection). (3) Pathological specimens deemed damaged, lost, or technically unsuitable for immunohistochemical (IHC) staining. (4) Participation in clinical trials or receipt of neoadjuvant therapy (chemotherapy or radiotherapy) between initial cancer diagnosis and the index surgery, to preclude confounding alterations to the tumor immune microenvironment. (5) Incomplete follow-up records.

Clinico-pathological data were extracted from the electronic medical records system. Variables collected included: patient age, sex, body mass index (BMI, kg/m^2^), smoking status, family history of colon cancer, tumor-node-metastasis (TNM) stage (AJCC/UICC), maximal tumor diameter (cm), tumor location, histological differentiation grade (well/moderate/poor), presence of vascular invasion, presence of lymphatic invasion, presence of perineural invasion, administration of adjuvant radiotherapy, administration of adjuvant chemotherapy. BMI was calculated using height and weight measured at hospital admission. Smoking status was defined as current or former smoking with a cumulative smoking index > 400 pack-years and cessation occurring within ≤2 years prior to colon cancer diagnosis. A positive family history required at least one second-degree relative diagnosed with colorectal cancer. Tumors localized to the cecum, ascending colon, hepatic flexure, or transverse colon were classified as proximal (right-sided), while tumors located in the splenic flexure, descending colon, or sigmoid colon were classified as distal (left-sided). Follow-up Protocol: Postoperative surveillance followed institutional protocols based on national clinical guidelines. Follow-up data collection methods included structured telephone interviews, review of inpatient admission records, and outpatient clinic visits. Active follow-up continued for a minimum of 5 years post-surgery, with scheduled assessments at approximately 3-month intervals. The primary endpoints analyzed were: Disease-Free Survival (DFS): Defined as the time interval from the date of initial pathologic diagnosis to the first documented event of locoregional recurrence, distant metastasis, death from any cause, or the date of the last follow-up contact (October 2021), whichever occurred first. Overall Survival (OS): Defined as the time interval from the date of initial pathologic diagnosis to death from any cause or the date of the last follow-up contact.

#### 2.1.2. Propensity Score Matching (PSM)

To minimize potential selection bias and imbalances in baseline characteristics between the obstruction group and controls, one-to-one propensity score matching (PSM) was performed without replacement. Potential confounding variables associated with tumor biology and prognosis in colorectal cancer—including age, sex, primary tumor location (proximal/distal), histological type, T stage, N stage, and preoperative Carcinoembryonic Antigen (CEA) level—were incorporated into the PSM model using logistic regression. The matching caliper was set to 0.2 times the standard deviation of the logit of the propensity score. Adequacy of matching was assessed by checking for standardized mean differences (SMD) < 0.1 for all included covariates and by comparing baseline characteristics after matching (using *t*-test, Mann–Whitney U, or Chi-square/Fisher as appropriate). Survival analyses (K-M and Cox) were repeated on the matched cohort.

### 2.2. Experimental Methods

#### 2.2.1. Tissue Analysis

Tissue Preparation: Formalin-fixed, paraffin-embedded (FFPE) tissue blocks containing representative tumor specimens were selected for analysis. Sections required ≥5% viable tumor cellularity within the central tumor (CT), presence of the tumor invasive margin (IM), and adjacent morphologically normal colonic mucosa. Immunohistochemistry (IHC): Serial tissue sections of 4-µm thickness were prepared. Deparaffinization, rehydration, and antigen retrieval were performed using Tris-EDTA buffer (pH 8.0). Endogenous peroxidase activity was quenched. Sections were incubated with specific primary antibodies diluted in antibody diluent at 37 °C for 45 min. The primary antibodies, dilutions, sources, and catalog numbers used were:

Anti-CD4: 1:500, ab133616 (Abcam, Cambridge, UK)

Anti-CD8: 1:2500, ab85792 (Abcam, Cambridge, UK)

Anti-CD20: 1:5000, 60271-1-Ig (Proteintech, Wuhan, China)

Anti-CD68: 1:6000, ab955 (Abcam, Cambridge, UK)

Following primary incubation and phosphate-buffered saline (PBS) washes, sections were incubated with appropriate horseradish peroxidase (HRP)-conjugated secondary antibodies at 37 °C for 30 min. Immunoreactivity was visualized using 3,3′-Diaminobenzidine (DAB) substrate (DAB IHC Detection Kit, Abcam, UK) for 10 min. Sections were counterstained with Mayer’s hematoxylin, dehydrated, and permanently mounted with resinous mounting medium. Whole-slide images (WSI) were digitally captured at 20× magnification (0.45 µm/pixel resolution) using a Nanozoomer slide scanner. An experienced pathologist reviewed all stained sections to confirm staining quality, specificity, and adequacy prior to quantitative analysis.

#### 2.2.2. QuPath Digital Image Analysis

Digitized WSI were imported into QuPath software (Version: 0.4.1, Queen’s University Belfast, Belfast, Northern Ireland, UK) for quantitative analysis [11]. Stain vectors were automatically estimated using optical density decomposition algorithms to standardize color separation. The tumor IM was manually delineated by a pathologist. A defined 500-µm wide continuous region was then annotated: extending 500 µm inwards from the IM into the tumor tissue and 500 µm outwards into the adjacent histologically normal mucosa. The CT was segmented by first creating the inverse of the entire tumor annotation (including IM) and then manually excluding areas of normal tissue, necrosis, or artifact. Automated quantification of positive immune cells was performed within the CT and IM zones using the integrated “Positive Cell Detection” plugin. Parameters (including intensity thresholds, cell size limits, and background subtraction) were optimized on a training set and kept consistent across all analyses. Cell counts for CD4^+^, CD8^+^, CD20^+^, and CD68^+^ cells were recorded for each region per patient sample.

### 2.3. Statistical Analysis

Statistical analyses were performed using IBM SPSS Statistics (version 29.0; Armonk, NY, USA), GraphPad Prism (version 7.0; San Diego, CA, USA), and R software (version 3.4.4; R Foundation for Statistical Computing, Vienna, Austria). A two-sided *p*-value of <0.05 was considered statistically significant.

Continuous variables were assessed for normality (e.g., using Shapiro–Wilk test). Normally distributed variables are presented as mean ± standard deviation (SD) and compared using Student’s *t*-test. Non-normally distributed variables are presented as median with interquartile range (IQR) and compared using the Wilcoxon rank-sum test (or Mann–Whitney *U*-test). Categorical variables are presented as frequencies and percentages and compared using the Chi-square test or Fisher’s exact test (where expected cell counts were <5). These analyses were applied to compare clinicopathological characteristics, laboratory values, tumor features, and surgical factors between the obstruction group and the non-obstruction control group. Boxplots were generated to visualize medians, IQRs, and outliers for continuous data.

#### 2.3.1. Multivariate Analysis

Variables demonstrating a univariate association with survival (typically *p* < 0.10) were considered for inclusion in multivariate Cox proportional hazards regression models. Models were constructed using backward stepwise selection (significance level for removal *p* ≥ 0.10) or enter methods, as appropriate. Multivariate analyses yielded adjusted HRs and 95% CIs to identify independent prognostic factors for OS and DFS. Assumptions of proportional hazards for Cox models were tested using Schoenfeld residuals.

#### 2.3.2. Survival Analysis

Survival functions for Disease-Free Survival (DFS) and Overall Survival (OS) were estimated using the Kaplan–Meier (K-M) method. Differences in survival between groups were evaluated using the log-rank test. Univariate Cox proportional hazards regression models were performed to calculate unadjusted hazard ratios (HR) and 95% confidence intervals (CI) for factors potentially associated with DFS and OS. Optimal cutoff points for stratifying patients based on continuous variables (e.g., immune cell densities, tumor markers) were determined using X-tile software, utilizing DFS data to identify maximally discriminative thresholds stratified by obstruction status. Associations between continuous variables were assessed using Spearman’s rank correlation coefficient (ρ).

## 3. Result

### 3.1. Patient Enrollment Screening and Clinical Baseline Data

**Figure 1:**
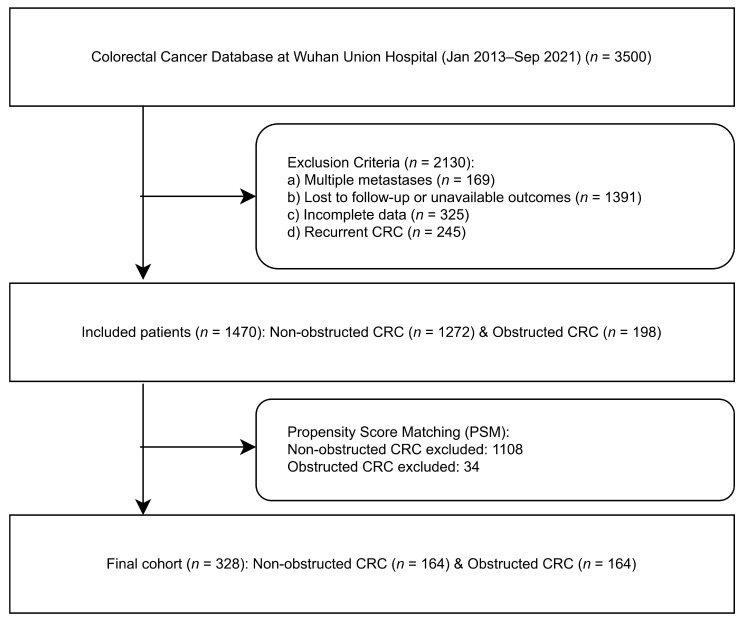
Enrollment flowchart. 164 obstructed [OCRC] vs. 164 non-obstructed [NOCRC] were recruited into this study after propensity score matching. *n* indicates the number of patients.

The study initially identified 3500 patients from the Colorectal Cancer Database at Wuhan Union Hospital (January 2013 to September 2021). After applying sequential exclusion criteria, 2130 cases (60.9%) were excluded: 169 patients with multiple metastases, 1391 with incomplete follow-up or unavailable outcomes, 325 with missing data, and 245 with recurrent colorectal cancer (CRC). This resulted in 1470 eligible patients, comprising 1272 non-obstructed CRC cases and 198 obstructed CRC cases. To mitigate potential confounding factors, propensity score matching (PSM) was performed, excluding 1108 non-obstructed and 34 obstructed CRC cases due to unmatched covariates. The final balanced cohort consisted of 328 patients (164 non-obstructed CRC and 164 obstructed CRC), ensuring comparable baseline characteristics between groups. This rigorous, stepwise screening process, illustrated in the enrollment flowchart (Figure 1), underscores methodological transparency and minimizes selection bias, thereby enhancing the validity of subsequent comparative analyses.

Baseline characteristics of the 328 patients included in the study cohort, stratified by obstruction status (non-obstructed, *n* = 164; obstructed, *n* = 164), are summarized in Table 1. No significant differences were observed between the two groups in age (non-obstructed: 62.2 ± 11.8 years vs. obstructed: 61.7 ± 13.3 years, *p* = 0.74), BMI (21.6 ± 3.0 kg/m^2^ vs. 22.0 ± 2.8 kg/m^2^, *p* = 0.21), or sex distribution (55.5% male vs. 56.1% male, *p* = 1.00). Clinical variables, including smoking history (18.3% vs. 24.4%, *p* = 0.23), comorbidities (32.9% vs. 32.3%, *p* = 1.00), prior abdominal surgery (25.0% vs. 22.0%, *p* = 0.60), cardiovascular disease (27.4% vs. 30.5%, *p* = 0.63), diabetes mellitus (7.9% vs. 8.5%, *p* = 1.00), and hematological disorders (0.6% vs. 0.6%, *p* = 1.00), were comparable between groups. Cerebrovascular disease was more prevalent in the obstructed group (3.7% vs. 0.6%, *p* = 0.12), though this difference did not reach statistical significance. Tumor staging revealed significant differences in TNM classification (*p* = 0.002). The obstructed group had a higher proportion of stage II (41.5% vs. 30.5%) and stage IV (7.3% vs. 2.4%) disease, whereas the non-obstructed group had more stage I (11.0% vs. 1.8%) and stage III (56.1% vs. 49.4%) cases. Perineural invasion was more frequent in the obstructed group (32.3% vs. 21.3%, *p* = 0.034). No significant differences were observed in T stage (*p* = 0.14), N stage (*p* = 0.74), M stage (*p* = 0.72), tumor differentiation (*p* = 0.94), lymphovascular invasion (*p* = 0.27), tumor size (*p* = 0.54), or tumor location (*p* = 0.79). A history of malignancy was more common in the non-obstructed group (11.0% vs. 4.9%, *p* = 0.066), though this trend was not statistically significant. ASA classification (*p* = 0.16) and rates of neoadjuvant therapy (3.7% vs. 3.0%, *p* = 1.00) did not differ between groups. The obstructed and non-obstructed groups were well-balanced in most baseline characteristics, except for TNM staging and perineural invasion, which showed statistically significant disparities. Following propensity score matching, well-balanced cohorts were achieved for most clinical covariates. However, as might be expected given the nature of the disease, slight differences in TNM stage and perineural invasion remained between the obstructed and non-obstructed groups. This likely reflects the intrinsic biological link between intestinal obstruction and more locally aggressive, advanced tumors. These factors were included as covariates in subsequent multivariate survival analyses to mitigate their potential confounding effects.

### 3.2. Digital Annotation of Tumor Whole Slide Images and Quantification of Tumor-Infiltrating Immune Cells

**Figure 2:**
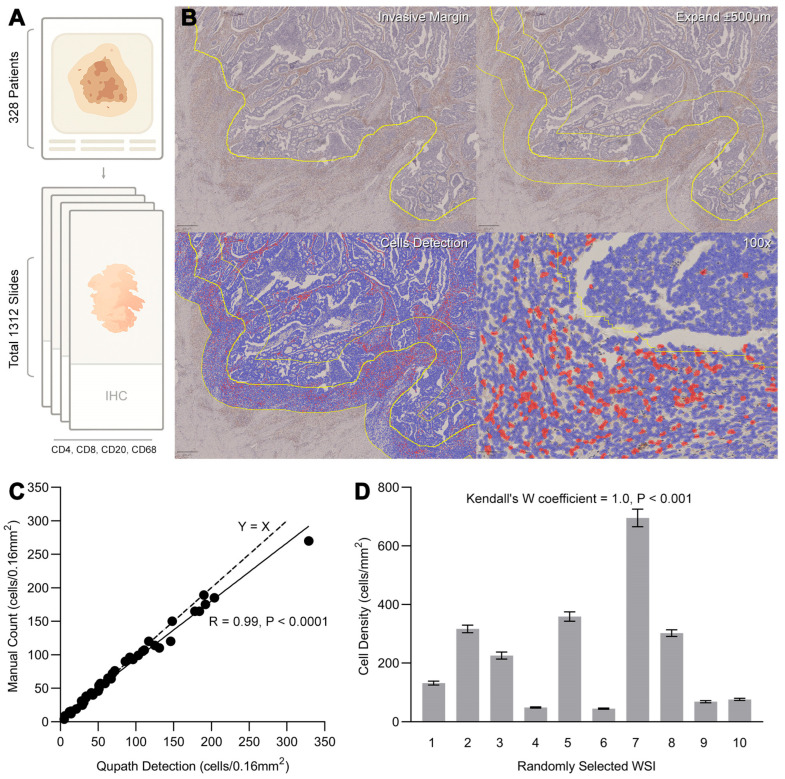
Digital Annotation of Tumor Whole Slide Images and Quantification of Tumor-Infiltrating Immune Cells (**A**) Representative IHC images stained for immune cell markers (CD4, CD8, CD20 and CD68) and whole-slide images scan. (**B**) Schematic of the quantification workflow using QuPath software. Detected positive immune cells are highlighted in red. (**C**) Scatter plot showing the correlation between manual and QuPath immune cell counts. QuPath quantification overcomes the omissions and inefficiencies of manual counting while maintaining extremely high consistency (R = 0.99, *p* < 0.0001). (**D**) Inter-operator agreement in IM region annotation and immune cell detection (Kendall’s W coefficient = 1.0, *p* < 0.001). IHC: Immunohistochemistry. WSI: Whole Slide Image. Qupath: Digital image analysis software (Version: 0.4.1, Queen’s University Belfast, Belfast, Northern Ireland, UK).

Surgical specimens underwent serial sectioning followed by standardized immunohistochemical (IHC) staining protocols and passed quality control review. A total of 1312 immunohistochemically stained pathological slides were evaluated by consensus among pathologists. Of these, 328 patients with complete sections for all four markers (CD4, CD8, CD20, CD68) were included in the subsequent immune cell infiltration analysis. Whole-slide images (WSI) were acquired using a Nanozoomer slide scanner (bright field, 20× magnification, 0.45 μm/pixel resolution), with manual focus selection and quality verification to ensure compliance with predefined standards for resolution and image clarity (Figure 2A).

Following a standardized QuPath protocol for tumor-infiltrating immune cell analysis (Figure 2B), trained operators annotated tumor IM on WSI. Annotation times ranged from 15 to 90 sec per slide initially, varying depending on tumor border complexity and size. With proficiency, IM annotation time was reduced to 5–15 sec per slide. For serial IHC sections, IM annotations from the first slide were replicated and adjusted (via rotation/translation) to align with subsequent slides, significantly optimizing workflow efficiency. After inverting the IM annotation, CT were delineated by manually erasing blank areas and normal tissue using the brush tool. Automated immune cell detection in QuPath, facilitated by custom scripts, exported immune cell density (cells/mm^2^) for each compartment (IM and CT).

To validate the accuracy of automated immune cell counting, 40 randomly selected WSI were analyzed. A 400 × 400 μm region within the IM was manually counted and compared with QuPath-automated detection. Results demonstrated strong concordance between manual and automated counts (R = 0.99, *p* < 0.0001; Figure 2C). Three non-expert operators (medical students) independently annotated IM regions on 10 randomly selected WSI. High consistency was observed across operators (Kendall’s W coefficient = 1.0, *p* < 0.001; Figure 2D), confirming the reproducibility of the QuPath-based workflow.

### 3.3. The Impact of Obstruction Status and T Stage on the Patterns of Tumor Immune Cell Infiltration

#### 3.3.1. Obstruction May Be Associated with High Lymphocyte Infiltration in Tumors, with T4 Tumors Exhibiting a Lymphocyte Infiltration Tendency Comparable to T1–3 Tumors

**Figure 3:**
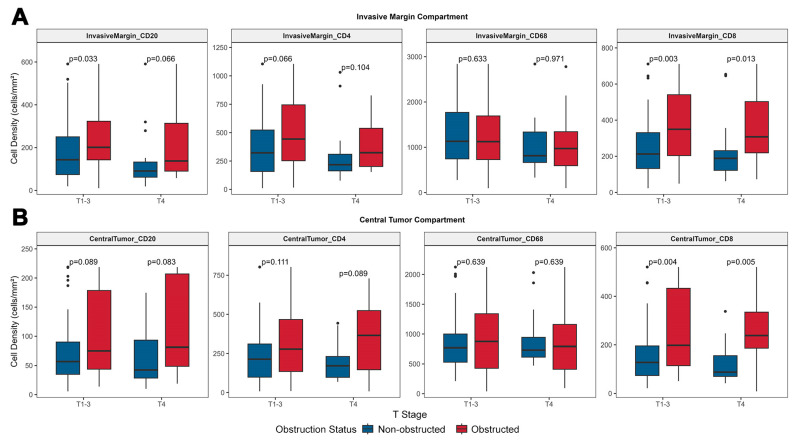
Immune Cell Density Distribution by Tumor Compartment and T Stage. Density of CD68 macrophages remains consistent across T stages and obstruction status. Independent of T stage, CD8^+^ T cells show a significant increase in OCRC (IM: *p* = 0.003 for T1–3, *p* = 0.013 for T4; CT: *p* = 0.004 for T1–3, *p* < 0.005 for T4), whereas the increases in CD4^+^ T cells and CD20^+^ B cells in OCRC are not statistically significant.

In the IM compartment, the densities of all three tumor-infiltrating lymphocytes (TILs) subsets were lower in T4 tumors compared to T1–3 tumors, irrespective of obstruction status, consistent with previous literature, although the observed differences in this study did not reach statistical significance (Figure 3A). Among OCRC, the densities of all three infiltrating lymphocyte subsets (IM-CD4^+^ T cells, IM-CD8^+^ T cells, and IM-CD20^+^ B cells) were higher than those in NOCRC (although only IM-CD8^+^ T cells showed statistical significance: *p* = 0.003 for T1–3 and *p* = 0.013 for T4, Figure 3A). In the CT compartment, a similar trend was observed in non-obstructed tumors, where T4 tumors exhibited lower densities of all three TILs subsets compared to T1–3 tumors (statistically non-significant, Figure 3B). However, in obstructed tumors, the opposite pattern emerged, with T4 tumors showing greater immune infiltration than T1–3 tumors. This was accompanied by higher densities of all three TILs subsets in obstructed tumors, although only CT-CD8^+^ T cells demonstrated statistical significance (*p* = 0.004 for T1–3 and *p* = 0.005 for T4). This phenomenon of relatively high lymphocyte infiltration in CT compartment of T4 tumors in the obstructed group suggests that obstruction may have differential effects on TILs between T1–3 and T4 tumors, with a more pronounced impact in T4 tumors. Notably, the infiltration of CD68^+^ macrophages in the IM and CT compartments showed no significant differences between T1–3 and T4 tumors, nor between the obstructed and non-obstructed groups. However, in the CT compartment, the density of CD68 of OCRC exhibited higher variability compared to the non-obstructed group.

#### 3.3.2. Compartmental and Inter-Subsets Correlations Reveal Distinct Patterns of the Tumor Immune Microenvironment Across Different T Stages and Obstruction Status

**Figure 4:**
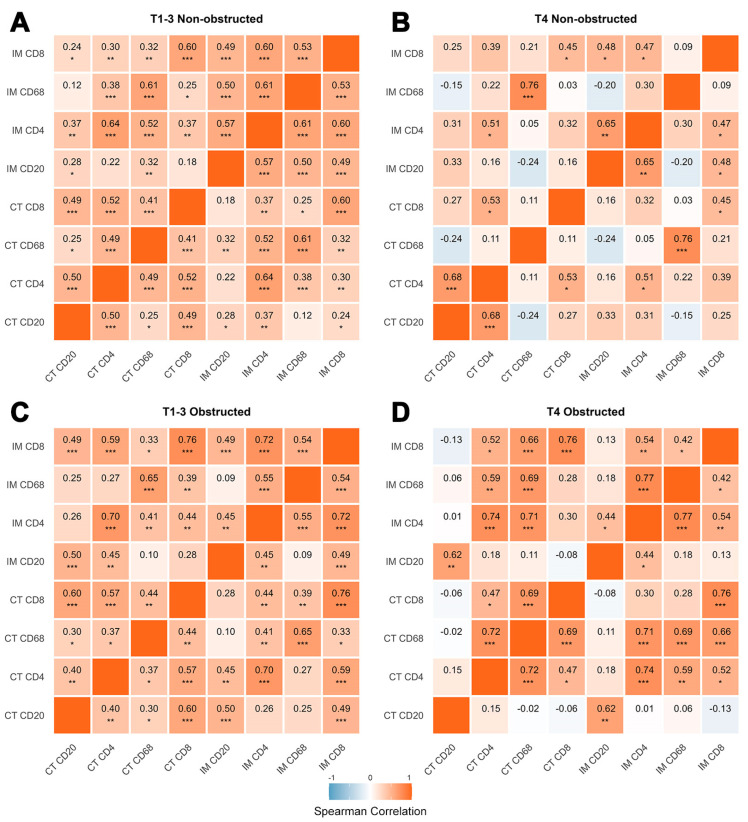
Immune Cell Marker Correlations by T Stage and Obstruction Status. Spearman correlation coefficients with significance: * *p* < 0.05, ** *p* < 0.01, *** *p* < 0.001. IM: Invasive Margin. CT: Central Tumor.

The positive correlations of four tumor-infiltrating immune cell subsets between the CT and IM compartment are influenced by T stage and obstruction status, with obstruction generally enhancing these correlations (Figure 4C,D). In the NOCRC, compared to T1–3 tumors, T4 tumors exhibited higher correlations for CD68^+^ macrophages between the CT and IM compartments but lower or statistically insignificant correlations for lymphocytes (0.76 vs. 0.61 for CD68, Figure 4B). In the obstructed group, T4 tumors showed higher compartmental correlations for infiltrating immune cells across all comparisons compared to T1–3 tumors (0.74 vs. 0.70 for CD4; 0.76 vs. 0.76 for CD8; 0.62 vs. 0.50 for CD20; 0.69 vs. 0.65 for CD68, Figure 4C,D). Considering that the low compartmental correlation in the non-obstructed group is mainly influenced by heterogeneity in both the tumor itself and the TIME, we propose that the increased compartmental correlations observed in the obstructed group imply that the emergence of a novel immune infiltration pattern has altered or masked the inherent immune infiltration characteristics.

T stage significantly affected correlations among different immune cell subsets, but the responses differed between obstructed and non-obstructed group (Figure 4). While positive correlations among various immune cell subsets were generally observed in T1–3 tumors, these correlations were largely reduced or absent in T4 tumors. However, in the non-obstructed group, T4 tumors showed enhanced positive correlations for CD4^+^ T cells and CD20^+^ B cells in the CT and IM compartments, respectively (0.68 vs. 0.50 for CT; 0.65 vs. 0.57 for IM, Figure 4B). In contrast, the obstructed group primarily exhibited enhanced positive correlations for CT-CD68^+^ macrophages with CD4^+^ and CD8^+^ T cells in both the CT and IM compartments of T4 tumors. Meanwhile, the high correlation between CT-CD68 and CD4^+^ and CD8^+^ T cells in both the CT and IM compartments of the obstructed group reveals that these elevated T cell populations are likely chemotactically recruited by macrophages in the CT. This suggests that these macrophages may play a central role in shaping obstruction-associated immune patterns. The low degree of correlation among immune cell subsets observed in T4-stage non-obstructed tumors enhances the contrasting effect induced by obstruction, thereby providing clearer insights into how obstruction modulates the tumor immune microenvironment. The pronounced compartmental correlations among immune cell subsets, as well as the significant associations between CD68 and CD4 or CD8 within the CT compartment observed in T4 tumors of the obstructed group, stand in stark contrast to the non-obstructed group, while the impact of obstruction on T3 tumors appears relatively limited. This suggests that obstruction may differentially affect tumors with active immune responses versus those exhibiting immune exhaustion, potentially explaining the disparities observed between T1–3 and T4 stage tumors.

#### 3.3.3. Immune Cell CT/IM Ratios Differentially Predict Colorectal Tumor Obstruction Status Across T Stages

**Figure 5:**
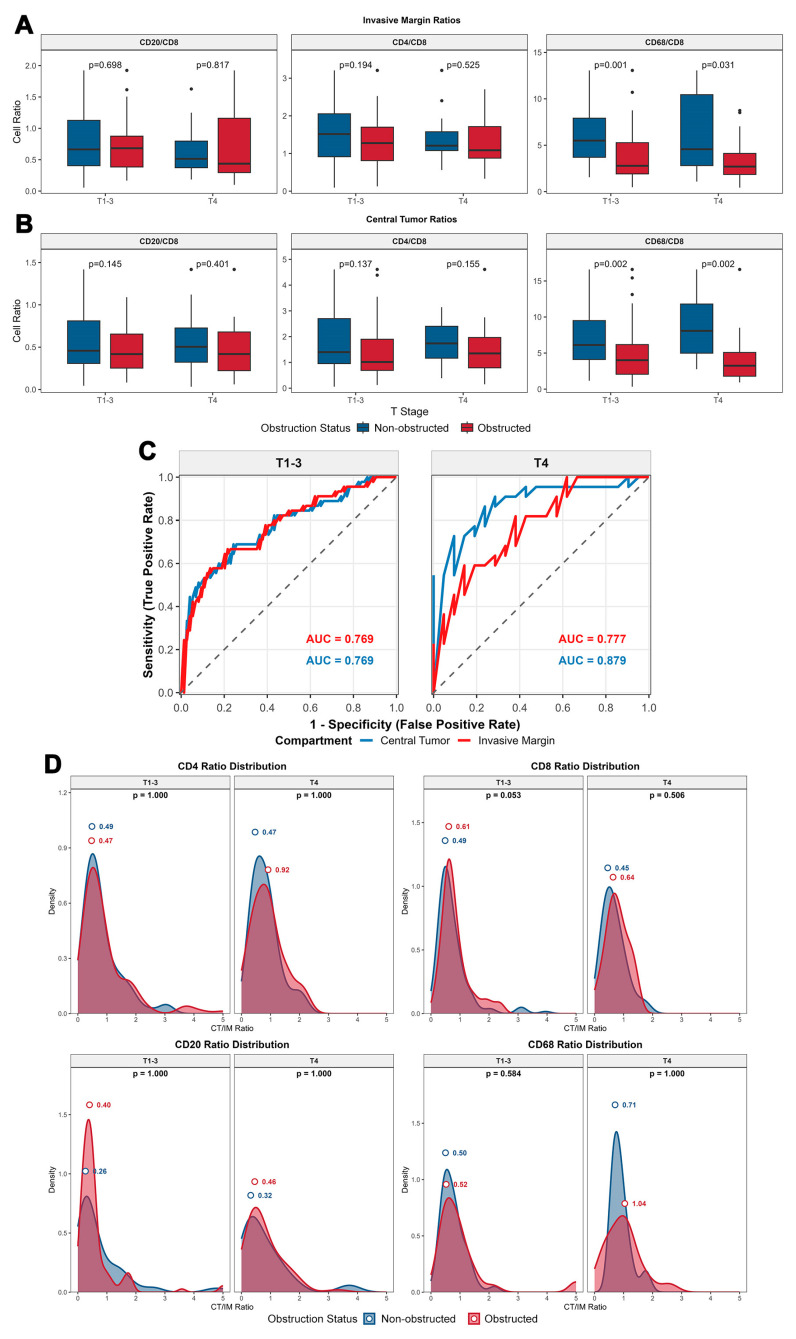
Compartmental and Inter-Subsets Correlations of immune cells. (**A**,**B**) Immune cell ratios by tumoral compartment and T stage. The CD68/CD8 ratio was significantly lower in the obstruction group than in the non-obstruction group in both the IM and CT compartments (IM: *p* = 0.001 for T1–3, *p* = 0.031 for T4; CT: *p* = 0.002 for T1–3, *p* = 0.002 for T4). (**C**) CD68^+^/CD8^+^ ratio predictive performance by T stage. The CD68+/CD8+ ratio accurately discriminated OCRC from NOCRC (T1–3: AUC = 0.769 for CT&IM; T4: AUC = 0.879 for CT; AUC = 0.777 for IM). (**D**) CT/IM ratio distribution by T stage and obstruction status. OCRC exhibited elevated CT/IM ratios for CD8+ T cells (0.61 vs. 0.49 for T1–3; 0.64 vs. 0.45 for T4), CD20^+^ B cells (0.40 vs. 0.26 for T1–3; 0.46 vs. 0.32 for T4), CD4^+^ T cells (0.92 vs. 0.47 for T4) and CD68^+^ macrophages (1.04 vs. 0.71 for T4), although the differences did not reach statistical significance. CD4, CD8, CD20, CD68: Immune Cell Markers. IM: Invasive Margin. CT: Central Tumor. T1–3, T4: Tumor Stage. AUC: Area Under the Receiver Operating Characteristic Curve. OCRC: Obstructed Colorectal Cancer. NOCRC: Non-Obstructed Colorectal Cancer.

Obstruction induces differences in the ratios of immune cell subsets, which can be used to pathologically distinguish tumor obstruction status. By calculating the ratios of various immune cells to CD8^+^ T cells, significant differences in the density ratios of CD68^+^ macrophages to CD8^+^ T cells were observed between obstructed and non-obstructed groups, independent of T stage (Figure 5A,B). ROC curves assessing the CD68/CD8 ratio’s ability to predict obstruction status (Figure 5C)—stratified by T1–3 and T4 stages—demonstrated robust predictive performance (T1–3: AUC = 0.769 for IM, AUC = 0.769 for CT; T4: AUC = 0.777 for IM, AUC = 0.879 for CT).

By calculating the CT/IM ratio of tumor-infiltrating immune cells (Figure 5D), CD8^+^ T cells and CD20^+^ B cells exhibited similar patterns: the peak distribution of the CT/IM ratio was higher in obstructed tumors than in non-obstructed tumors (obstructed vs. non-obstructed: 0.61 vs. 0.49 for CD8 & T1–3; 0.64 vs. 0.45 for CD8 & T4; 0.40 vs. 0.26 for CD20 & T1–3; 0.46 vs. 0.32 for CD20 & T4). This difference was independent of T stage. In contrast, CD4^+^ T cells and CD68^+^ macrophages exhibited distinct distribution patterns: the peak CT/IM ratio in OCRC was significantly higher than that in Non-obstructed CRC in T4 tumor (obstructed vs. non-obstructed: 1.04 vs. 0.71 for CD68; 0.92 vs. 0.47 for CD20), whereas no such difference was observed in the T1–3 tumor (obstructed vs. non-obstructed: 0.52 vs. 0.50 for CD68; 0.47 vs. 0.49 for CD4). Although these differences did not reach statistical significance (*p* > 0.05), the results suggest that tumor-infiltrating immune cells exhibit a pattern of consistent infiltration across the entire tumor in OCRC, with no evidence of immune exclusion.

### 3.4. Peripheral Blood Immune Cell Profiles Are Compared Between OCRC and NOCRC Cases

**Figure 6:**
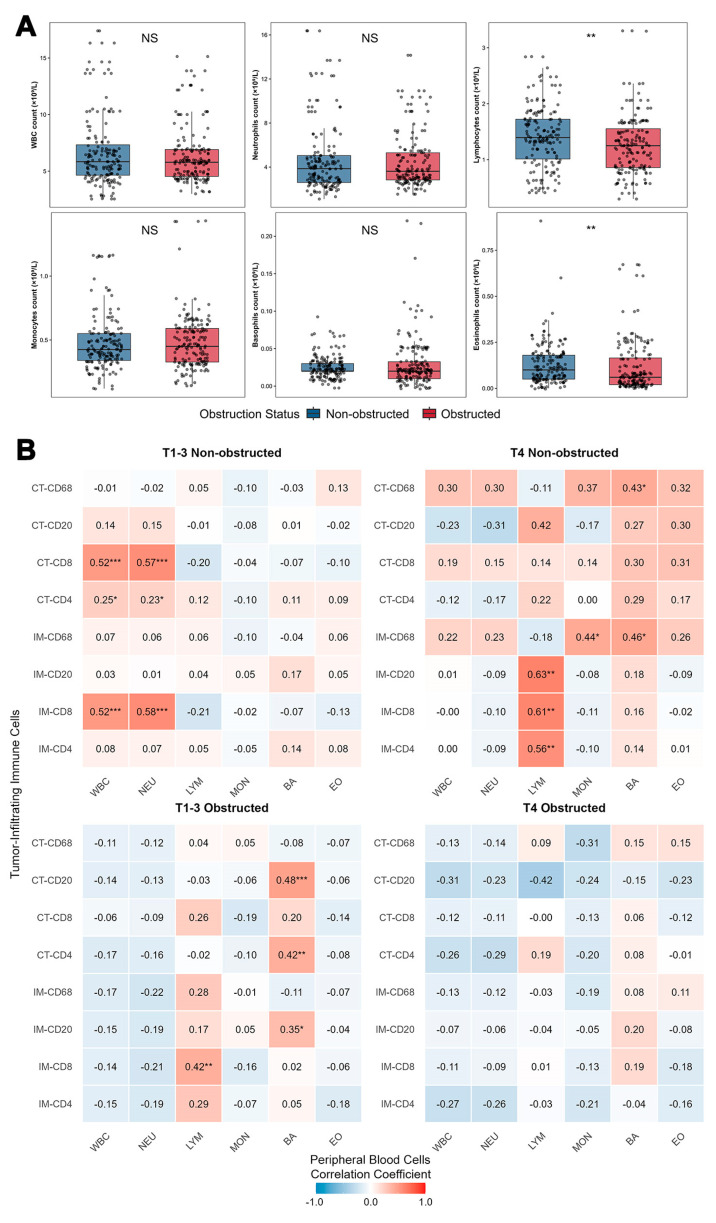
Systemic–Local Immune Crosstalk. (**A**) Peripheral Immune Cell Count by T Stage and Obstruction Status. Obstruction was associated with a non-significant decrease in lymphocyte counts, independent of T stage. (**B**) Correlation heatmaps of systemic–local immune cell. Obstruction significantly weakened the correlation between peripheral blood immune cells and tumor-infiltrating immune cells. * *p* < 0.05, ** *p* < 0.01, *** *p* < 0.001. NS: No Significance. CD4, CD8, CD20, CD68: Immune Cell Markers. IM: Invasive Margin. CT: Central Tumor. T1–3, T4: Tumor Stage. WBC: White Blood Cell (Count). NEU: Neutrophil (Count). LYM: Lymphocyte (Count). MON: Monocyte (Count). EO: Eosinophil (Count). BA: Basophil (Count).

No statistically significant differences were observed in the counts of peripheral blood white blood cells (WBC), neutrophils (NEU), monocytes (MON), or basophils (BA) between the two groups (*p* > 0.05, Figure 6A). However, patients with obstruction exhibited significantly lower LYM compared to those without obstruction (median: 1.25 vs. 1.40 × 10^9^/L, *p* = 0.003; mean: 1.26 ± 0.54 vs. 1.41 ± 0.55 × 10^9^/L). Similarly, eosinophil counts (EO) were significantly reduced in the obstruction group (median: 0.06 vs. 0.10 × 10^9^/L, *p* = 0.004; mean: 0.12 ± 0.14 vs. 0.13 ± 0.11 × 10^9^/L). Subgroup analyses revealed that the association between obstruction and immune profiles in CRC patients varied by T stage (Table 2). In the T1–3 subgroup, obstruction was associated with significantly lower eosinophil counts (0.11 ± 0.14 vs. 0.12 ± 0.09 × 10^9^/L, *p* = 0.005), but no significant differences were observed in other immune cells. Conversely, among T4 patients, those with obstruction had markedly reduced lymphocyte counts (1.22 ± 0.42 vs. 1.55 ± 0.48 × 10^9^/L, *p* < 0.001) and a trend toward higher monocyte counts (0.53 ± 0.28 vs. 0.42 ± 0.10 × 10^9^/L, *p* = 0.057), with no significant differences in other cell types. Detailed subgroup analyses are provided in Supplementary Tables S1–S9.

The obstruction status significantly altered the relationship between tumor immune infiltration and peripheral blood immune cells, with distinct patterns emerging across T stage and obstruction subgroups (Figure 6B). In non-obstructed T1–3 tumors, CD8^+^ T lymphocyte densities in both the IM and CT compartments showed strong positive correlations with peripheral blood NEU and WBC. Additionally, CT-CD4^+^ T lymphocyte density was positively correlated with NEU. These findings suggest that in earlier-stage, non-obstructed tumors, a systemic neutrophilic response may be coupled with cytotoxic T cell infiltration. In obstructed T1–3 tumors, these relationships were absent. Instead, IM-CD8^+^ T lymphocyte density showed a positive correlation with peripheral blood LYM. Significant positive correlations also emerged between both IM and CT compartment CD20^+^ B lymphocyte densities, as well as CT-CD4^+^ T lymphocyte density, with peripheral blood BA. This shift suggests that obstruction may alter local immune recruitment, potentially enhancing basophil and lymphocyte crosstalk.

In non-obstructed T4 tumors, correlations were observed primarily in the IM compartment: CD4^+^, CD8^+^, and CD20^+^ lymphocyte densities all correlated positively with peripheral blood LYM. CD68^+^ macrophage density in the IM correlated with MON, and both IM and CT CD68^+^ macrophage densities correlated with BA. No significant correlations were found between any CT compartment lymphocytes and peripheral parameters, indicating that in advanced but non-obstructed tumors, the IM may be the primary interface for systemic immune engagement. In obstructed T4 tumors, no significant correlations were detected between any tumor-infiltrating immune cell subset (in either IM or CT) and peripheral blood immune parameters. This complete decoupling suggests that mechanical obstruction and advanced T-stage may jointly disrupt systemic anti-tumor immune communication, potentially contributing to immunosuppression and disease progression. All other unmentioned correlations were non-significant (*p* > 0.05).

In summary, among CRC patients, obstructive status—particularly when combined with advanced T4 stage—significantly alters both the systemic and local tumor immune landscape, leading to a disruption in the coordinated relationship between the two. Patients with obstruction exhibit significantly lower peripheral blood LYM and EO compared to those without obstruction, with lymphocytopenia being especially pronounced in T4 patients with obstruction. Obstruction disrupts the association between tumor immune infiltration and systemic immune status, an effect that is most extreme in T4 tumors. While in non-obstructive T4 tumors, the densities of various immune cells (CD4^+^ T, CD8^+^ T, CD20^+^ B cells) in the IM remain positively correlated with peripheral blood lymphocyte counts (indicating preserved communication between local and systemic immunity), in obstructive T4 tumors, all correlations between tumor-infiltrating immune cell subsets (both in IM and CT) and peripheral blood immune parameters are completely absent. This suggests the occurrence of a "systemic–local immune decoupling."

Based on these findings, obstruction-associated hyperinfiltration of tumor-infiltrating lymphocytes is unlikely to represent a secondary effect of systemic immune hyperactivation. Instead, it may occur within a context of systemic immunosuppression, as evidenced by peripheral lymphocytopenia, particularly in T4 patients with obstruction. The complete disruption of systemic–local immune coordination observed in obstructed T4 tumors further supports this notion, indicating a decoupling between local immune activity and systemic immune status. These findings provide a compelling rationale for stratifying immunotherapy strategies based on both obstruction status and T stage, thereby modifying the prognostic interpretation of TIL assessments.

### 3.5. Impact of Obstruction and Tumor Immune Cell Infiltration on Patient Survival

#### 3.5.1. Prognostic Factors in the Full Cohort

Univariate and multivariate Cox regression analyses (Table 3 and Table 4) identified consistent predictors of disease-free survival (DFS) and overall survival (OS). In univariate analysis, advanced T stage (T3, T4), nodal involvement (N1–2), distant metastasis (M1), open surgical approach, blood transfusion, and nerve invasion were significantly associated with worse DFS and OS. Conversely, primary anastomosis and higher CD68^+^ macrophage density at the IM or CT correlated with improved outcomes. Multivariate analysis confirmed advanced T stage (T3: HR = 2.02, *p* = 0.047; T4: HR = 3.39, *p* = 0.001 for DFS; T4: HR = 3.29, *p* = 0.004 for OS), blood transfusion (DFS HR = 1.65, *p* = 0.022; OS HR = 1.92, *p* = 0.005), and lack of primary anastomosis (DFS HR = 0.53, *p* = 0.007; OS HR = 0.54, *p* = 0.010) as independent risk factors. CD68^+^ density at the IM remained a robust protective factor in both DFS (HR = 0.59, *p* = 0.008) and OS (HR = 0.60, *p* = 0.024), while its significance in the CT compartment was lost after adjustment. Notably, nerve invasion and M stage, significant in univariate analysis, were not retained in multivariate models.

#### 3.5.2. NOCRC Subgroup Analysis

In NOCRC patients (Table 5 and Table 6), age, advanced T stage (T3: HR = 6.59, *p* = 0.012; T4: HR = 10.1, *p* = 0.003), and M1 stage (HR = 6.70, *p* = 0.018) independently predicted poorer DFS. For OS, age (HR = 1.05/year, *p* = 0.017), T stage (T3: HR = 4.60, *p* = 0.044; T4: HR = 7.24, *p* = 0.012), and N1–2 stage (HR = 2.53, *p* = 0.044) were significant. Primary anastomosis showed a protective trend in both DFS (HR = 0.50, *p* = 0.068) and OS (HR = 0.50, *p* = 0.092), though not statistically significant. Immune markers (CD4^+^, CD8^+^, CD20^+^, CD68^+^) demonstrated prognostic value in univariate analysis (e.g., IM-CD20^+^: OS HR = 0.50, *p* < 0.001) but lost significance in multivariate models, suggesting their association may be confounded by clinicopathological factors.

#### 3.5.3. Dichotomous Role of CD8^+^ T Cells by Obstruction Status

**Figure 7:**
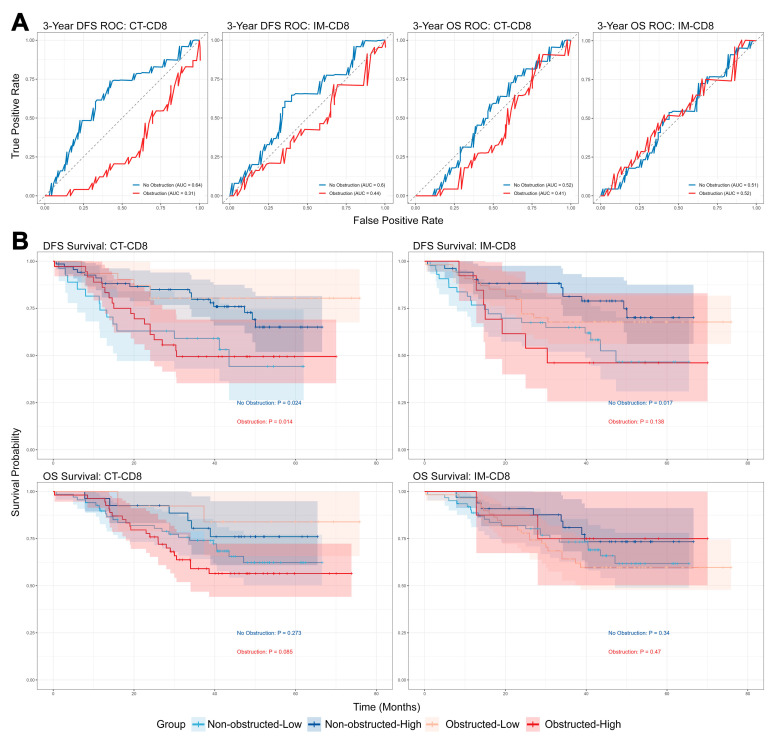
Impact of Obstruction and Tumor Immune Cell Infiltration on Patient Survival. (**A**) ROC curves assessing the prognostic value of CD8^+^ T cell density for survival outcomes in colorectal cancer patients stratified by obstruction status (Higher marker values predict better outcomes). ROC analysis for CD8^+^ T cell density in the CT predicting 3-year DFS. Non-obstructed cases (blue line): AUC = 0.64. Obstructed cases (red line): AUC = 0.69. (**B**) Kaplan–Meier survival analysis stratified by optimal CD8^+^ T cell density thresholds identified through X-tile in colorectal cancer patients with or without obstruction. DFS: Disease-Free Survival. OS: Overall Survival. CD8: Immune Cell Marker. IM: Invasive Margin. CT: Central Tumor. ROC: Receiver Operating Characteristic. AUC: Area Under the ROC Curve. Obstructed/Obstruction and Non-Obstructed/No Obstruction: Obstruction Status of CRC. High: Density above the optimal threshold. Low: Density below the optimal threshold.

Stratification by obstruction status revealed opposing prognostic effects of CD8^+^ T cell density (Figure 7A). In NOCRC, high CD8^+^ density in the IM and CT compartments was protective (3-year DFS AUC = 0.64 for CT-CD8), whereas in OCRC, it correlated with worse outcomes. Optimal cutoff values derived via X-tile further underscored this dichotomy (Figure 7B), highlighting the context-dependent role of CT-CD8^+^ cells. This paradoxical prognostic effect suggests that the extent of elevated lymphocyte infiltration induced by obstruction may be influenced by the pre-existing local immune context. Specifically, tumors with poorer prognosis appear to exhibit more pronounced obstructive effects, potentially enabling tumors that would otherwise show low lymphocyte infiltration to manifest heightened levels. This inferred mechanism aligns with the differential responses to obstruction observed between T4 and T3 stage tumors mentioned above, especially when considering that previous studies have often associated CD8+ T lymphocyte infiltration with improved prognosis. Consequently, CT-CD8 cell density may serve as a useful biomarker for assessing this “obstruction effect.”

Advanced tumor stage, blood transfusion, and lack of primary anastomosis were consistently independent predictors of poorer survival. CD68^+^ macrophages at the IM emerged as a stable protective factor that was independent of obstruction status, while immune markers exhibited univariate significance but lacked independence in multivariate models. The paradoxical association of CD8^+^ cells with prognosis underlines the need for obstruction-status-specific risk stratification.

## 4. Discussion

CRC remains a leading cause of cancer-related mortality worldwide, with obstruction representing a common and clinically significant complication in advanced disease [12]. While the TIME has been increasingly recognized as a critical determinant of prognosis and therapeutic response, the specific impact of mechanical obstruction on immune architecture and function remains poorly understood. To address this, we conducted a preliminary investigation that sought to delineate the immune landscape characterizing OCRC.

Obstruction in CRC, particularly in T4 tumors, is associated with distinct immune patterns, including enhanced CD8^+^ T cell infiltration, macrophage–lymphocyte coordination, and spatial redistribution of immune cells toward the tumor core. These findings underscore the importance of mechanical and pathological contexts in shaping the TIME and offer potential biomarkers for obstruction. The increased density of CD8^+^ T cells in the IM and CT compartments of OCRC, particularly in T4 tumors, suggests that mechanical obstruction may alter the immune landscape by promoting lymphocyte recruitment. This is consistent with studies indicating that local pathological stressors (e.g., hypoxia, necrosis, or bacterial overgrowth due to obstruction) can induce chemokine secretion (e.g., CXCL9/10) and enhance T-cell trafficking [13,14]. However, the lack of significant differences in CD4^+^ T cells and CD20^+^ B cells underscores the selective nature of this immune modulation, potentially driven by specific chemokine-chemokine receptor interactions (e.g., CXCR3 on CD8^+^ T cells). The enhanced correlations between immune cell subsets (e.g., CT-CD68^+^ macrophages with CT-CD4^+^ and CT-CD8^+^ T cells) in obstructed T4 tumors suggest that obstruction may foster a coordinated immune response primarily mediated by macrophages. Macrophages are known to recruit T cells via chemokines such as CCL5 and CCL22, and their spatial coupling with lymphocytes in OCRC aligns with studies describing macrophage-driven T-cell chemotaxis in other cancer types [15,16]. Conversely, the low inter-subset correlations in non-obstructed T4 tumors may reflect immune dysregulation typical of advanced CRC, characterized by stromal barriers, dysfunctional vasculature, or immunosuppressive factors (e.g., TGF-β, IL-10) [17]. The robust performance of the CD68^+^/CD8^+^ ratio in predicting obstruction status (AUC > 0.75 across T stages) highlights the biological relevance of macrophage–T-cell balance in obstructed tumors. A high ratio may indicate macrophage-dominated immunosuppression (e.g., M2 polarization) or, alternatively, inflammatory macrophage activation (e.g., M1-like) driving T-cell recruitment [18]. This dual role of macrophages aligns with their context-dependent functions in CRC, where M1 macrophages correlate with improved survival, while M2 macrophages associate with worse outcomes. The ratio’s predictive power suggests its utility as a pathological indicator of obstruction, though further validation is needed to clarify its functional implications. The elevated CT/IM ratios for CD8^+^ T cells, CD20^+^ B cells, and CD68^+^ macrophages in OCRC indicate a shift toward immune cell enrichment in the tumor core, contrary to the typical "immune-excluded" phenotype seen in advanced CRC. This pattern may result from obstruction-induced breakdown of architectural barriers (e.g., disrupted basement membranes) or upregulation of homing molecules (e.g., ICAM-1) facilitating immune cell penetration. Although not statistically significant, this trend echoes findings in other inflammation-driven cancers (e.g., microsatellite-instability-high CRC), where immune infiltration into the core correlates with improved responses to immunotherapy.

Obstruction is also associated with systemic immunosuppression (lymphocytopenia, eosinophil reduction) and disrupted systemic–local immune crosstalk. This decoupling suggests that the hyperinfiltration of TIICs in OCRC occurs within an immunosuppressive systemic context, rather than reflecting systemic immune activation. These findings underscore the need to integrate obstruction status and T-stage into immune-based prognostic models and therapeutic strategies for CRC. The observed reduction in peripheral LYM and EO in OCRC aligns with studies indicating that advanced CRC often induces systemic immune dysfunction. Specifically, lymphocytopenia is a known marker of immunosuppression in cancer and correlates with poor prognosis in multiple malignancies. Eosinophil depletion further supports an immunosuppressive phenotype, as eosinophils contribute to anti-tumor immunity via cytokine release (e.g., IL-4, IL-5) and direct cytotoxicity. The divergent effects of obstruction on peripheral immune cells across T stages highlight the complex interplay between tumor burden and local pathophysiology. In T1–3 tumors, obstruction primarily reduced EO without significantly affecting LYM, whereas in T4 tumors, obstruction was associated with pronounced lymphocytopenia and a trend toward MON. This suggests that advanced T-stage may amplify obstruction-induced immunosuppression. Monocytosis may indicate increased recruitment of myeloid-derived suppressor cells (MDSCs) or tumor-associated macrophages (TAMs), which are known to promote immune evasion in CRC [19]. These findings resonate with studies showing that T4 tumors exhibit more profound systemic immune dysregulation than earlier-stage lesions. The most striking finding is the complete loss of correlation between tumor-infiltrating immune cells (TIICs) and peripheral immune parameters in obstructed T4 tumors. In non-obstructed T4 tumors, immune densities in the IM remained correlated with peripheral LYM, suggesting preserved communication between local and systemic immunity. In contrast, obstructed T4 tumors exhibited a “systemic–local immune decoupling”, where TIICs were entirely disconnected from peripheral immune cell counts. This phenomenon aligns with studies highlighting the role of tumor-induced immune isolation in advanced CRC, where the TIME becomes metabolically and immunologically autonomous [19]. From a clinical perspective, this systemic–local immune decoupling suggests that for patients with advanced, obstructed tumors, the peripheral blood immune count may be a poor reflector of the local anti-tumor immune response. Consequently, clinical decisions regarding immunotherapy may need to rely more heavily on direct analysis of the tumor tissue itself, for example, through biomarker analysis of biopsy specimens.

Obstruction reconfigures the prognostic significance of tumor immune infiltration, neutralizing the protective role of CD8^+^ T cells while preserving the benefits of IM-CD68+ macrophages. The consistent association between higher IM-CD68+ density and improved survival (DFS HR = 0.59, OS HR = 0.60) across multivariate models underscores the potential anti-tumor role of macrophages in CRC. This contrasts with studies linking TAMs to immunosuppression and progression but aligns with evidence that macrophages can exhibit phenotypic plasticity and context-dependent functions [20,21]. In non-obstructed microenvironments, CD68^+^ cells may represent M1-polarized macrophages promoting phagocytosis, antigen presentation, and T-cell activation [22,23]. Their retention as an independent prognostic factor suggests that macrophage functional status—rather than mere density—may determine outcomes. Notably, the loss of significance for CT-CD68+ in multivariate analysis highlights the spatial importance of immune infiltration, with the IM serving as a critical interface for immune–tumor crosstalk [20]. The paradoxical prognostic impact of CD8^+^ T cells—protective in NOCRC (AUC = 0.64 for CT-CD8^+^) but adverse in OCRC—likely signifies a fundamental shift in their functional state. This contrasts sharply with the favorable, immunosurveillance-associated CD8^+^ infiltration characteristic of MSI-H tumors, which are driven by high neoantigen burden and are typically non-obstructive. In NOCRC, high CD8+ density likely signifies effective cytotoxic T-cell infiltration and immunosurveillance, consistent with established literature [24]. We hypothesize that the infiltrating CD8^+^ T cells in OCRC may be in a dysfunctional or exhausted state. In the setting of obstruction, microenvironmental stressors like hypoxia, necrosis, and potential bacterial translocation due to luminal stagnation could act as persistent antigenic stimuli [22,25]. Chronic antigen exposure is a known driver of T-cell exhaustion, a state characterized by progressive loss of effector function and high expression of inhibitory receptors such as PD-1, TIM-3, and LAG-3. This hypothesis could explain the limited efficacy of immune checkpoint inhibitors in microsatellite-stable CRC and suggests that obstructed patients might not benefit from PD-1/PD-L1 monotherapy despite having a high density of CD8^+^ T cells. Future studies incorporating single-cell RNA sequencing and exhaustion markers are essential to validate this hypothesis and to determine whether combinatorial strategies targeting alternative immune checkpoints or the exhausted T cell state itself could be beneficial for this patient subgroup. Multivariate models confirmed T stage, blood transfusion, and lack of primary anastomosis as independent predictors of poor survival, consistent with prior studies. The attenuation of immune cell prognostic value (e.g., CD20^+^ B cells, CD4^+^ T cells) in NOCRC multivariate models suggests their effects are confounded by clinicopathological covariates. This does not diminish their biological role but highlights the need for integrated prognostic models combining immune and conventional factors. Obstruction status should be incorporated into immune-based prognostic frameworks (e.g., Immunoscore adaptations). CD68^+^ density at the IM could serve as a robust biomarker for risk stratification. The adverse effect of CD8^+^ cells in OCRC may explain limited responses to immunotherapy in obstructed patients. Strategies to mitigate obstruction-related immunosuppression (e.g., TGF-β inhibition, CSF1R targeting) warrant exploration.

Our characterization of the distinct immune landscape in OCRC raises important questions regarding its association with therapeutic responses. Beyond the potential implications for ICIs discussed above, the reconfigured TIME of OCRC may also influence the efficacy of conventional and other targeted therapies. The obstruction-associated pathophysiology, including ischemia, altered stromal architecture, and bacterial overgrowth, could impair drug delivery and create a sanctuary site for tumor cells, potentially reducing the effectiveness of cytotoxic chemotherapy. Furthermore, the observed systemic–local immune decoupling and systemic immunosuppression (e.g., lymphocytopenia) might compromise the activity of systemically administered agents that rely on host immunity, such as certain chemotherapies whose efficacy is partly immunogenic. The spatial redistribution of immune cells towards the tumor core, while potentially enhancing local immune engagement, occurs within a broader immunosuppressive context that may necessitate combination strategies. These could include therapies targeting obstruction-related pathways, such as hypoxia (e.g., HIF inhibitors) or bacterial-driven inflammation (e.g., TLR4 antagonists), to resensitize the tumor microenvironment. Therefore, assessing obstruction status may provide critical insights for personalizing a broader range of therapeutic interventions in CRC.

Key limitations include the limited subgroup power for T4 obstructed cases (*n* = 54 after PSM), the restricted panel of immune markers evaluated (CD4, CD8, CD20, CD68), which prevents comprehensive immune phenotyping and functional assessment. Furthermore, the lack of systematic microsatellite instability (MSI) status data, coupled with unmeasured mechanistic drivers like hypoxia or microbiome shifts, limits our ability to fully contextualize the observed immune patterns within the known CRC immunogenetic framework. Notably, the absence of T-cell exhaustion markers (e.g., PD-1, TIM-3, LAG-3, TIGIT) limits our ability to conclusively characterize immune exhaustion states. Future work should validate findings in multi-ethnic cohorts with standardized obstruction criteria, interrogate immune dysfunction mechanisms via single-cell RNA sequencing to dissect CD8^+^ T-cell states [26], and explore TLR4 antagonists to mitigate potential bacterial-driven inflammation in obstructed CRC [27]. The diagnostic performance of CD68/CD8 ratios also requires prospective validation in biopsy specimens prior to surgical intervention. Longitudinal studies assessing immune changes pre-/post-decompression could clarify causal links between obstruction and immune alteration.

## 5. Conclusions

Based on our initial findings, this study provides a preliminary characterization of how obstruction status, particularly in T4-stage tumors, may influence the immunological landscape of CRC. Our results suggest that obstruction could represent more than a mechanical complication—potentially acting as an immunologically active state that might contribute to distinct patterns of immune cell infiltration, spatial redistribution, and altered systemic–local immune communication. These observed differences indicate that conventional interpretations of immune biomarkers may require further refinement and highlight potential complexities in CRC immunobiology that warrant deeper investigation. By integrating multi-compartment immune profiling with clinical outcomes, our data lend initial support to the idea that obstruction could help shape a specific immune contexture, which might have implications for prognostic stratification and therapeutic strategies. However, these insights are exploratory and will require validation in larger, independent cohorts. If confirmed, they highlight the need for tissue-based rather than blood-based immune profiling to guide therapy in patients with obstructed CRC.

## Figures and Tables

**Table 1:** Baseline Characteristics of Study Cohort.

		Study Cohort			Statistical Analysis
	Variable	Total (*n* = 328)	Non-Obstructed (*n* = 164)	Obstructed (*n* = 164)	*p* Value
Demographics	Age (years)	62.0 ± 12.6	62.2 ± 11.8	61.7 ± 13.3	0.74
	BMI (kg/m^2^)	21.8 ± 2.9	21.6 ± 3.0	22.0 ± 2.8	0.21
	Male	183 (55.8)	91 (55.5)	92 (56.1)	1
Clinical Characteristics	Smoking	70 (21.3)	30 (18.3)	40 (24.4)	0.23
	Comorbidity	107 (32.6)	54 (32.9)	53 (32.3)	1
	Prior abdominal surgery	77 (23.5)	41 (25.0)	36 (22.0)	0.6
	Cardiovascular disease	95 (29.0)	45 (27.4)	50 (30.5)	0.63
	COPD	14 (4.3)	9 (5.5)	5 (3.0)	0.41
	Diabetes mellitus	27 (8.2)	13 (7.9)	14 (8.5)	1
	Cerebrovascular disease	7 (2.1)	1 (0.6)	6 (3.7)	0.12
	Hematological disorders	2 (0.6)	1 (0.6)	1 (0.6)	1
Tumor Profile	T stage				0.14
	T1	9 (2.7)	4 (2.4)	5 (3.0)	
	T2	47 (14.3)	25 (15.2)	22 (13.4)	
	T3	182 (55.5)	99 (60.4)	83 (50.6)	
	T4	90 (27.4)	36 (22.0)	54 (32.9)	
	N stage				0.74
	N0	147 (44.8)	70 (42.7)	77 (47.0)	
	N1	123 (37.5)	64 (39.0)	59 (36.0)	
	N2	58 (17.7)	30 (18.3)	28 (17.1)	
	M stage				0.72
	M0	320 (97.6)	161 (98.2)	159 (97.0)	
	M1	8 (2.4)	3 (1.8)	5 (3.0)	
	TNM				**0.002**
	I	21 (6.4)	18 (11.0)	3 (1.8)	
	II	118 (36.0)	50 (30.5)	68 (41.5)	
	III	173 (52.7)	92 (56.1)	81 (49.4)	
	IV	16 (4.9)	4 (2.4)	12 (7.3)	
	Differentiation				0.94
	Low		24 (14.5)	26 (15.8)	
	Middle		117 (70.9)	114 (69.1)	
	High		23 (13.9)	24 (14.5)	
	Other		1 (0.6)	1 (0.6)	
	Perineural invasion	88 (26.8)	35 (21.3)	53 (32.3)	**0.034**
	Lymphovascular invasion	65 (19.8)	28 (17.1)	37 (22.6)	0.27
	Tumor Size				0.54
	<2 cm	13 (4.0)	5 (3.0)	8 (4.9)	
	2–5 cm	222 (67.7)	115 (70.1)	107 (65.2)	
	≥5 cm	93 (28.4)	44 (26.8)	49 (29.9)	
	Tumor Location				0.79
	Right	112 (34.1)	58 (35.4)	54 (32.9)	
	Left	132 (40.2)	63 (38.4)	69 (42.1)	
	Rectum	84 (25.6)	43 (26.2)	41 (25.0)	
	ASA				0.16
	I	3 (0.9)	2 (1.2)	1 (0.6)	
	II	214 (65.2)	100 (61.0)	114 (69.5)	
	III	65 (19.8)	40 (24.4)	25 (15.2)	
	IV	46 (14.0)	22 (13.4)	24 (14.6)	
Medical History	History of malignancy	26 (7.9)	18 (11.0)	8 (4.9)	0.066
	Neoadjuvant Therapy	11 (3.4)	6 (3.7)	5 (3.0)	1

Baseline Characteristics. Continuous variables as mean ± standard deviation (SD); Categorical variables as number of patients (*n*) with percentage (%). *t*-test for continuous variables, χ^2^/Fisher’s exact test for categorical variables. *p* less than 0.05 were considered statistically significant and are shown in bold. BMI: Body Mass Index. COPD: Chronic Obstructive Pulmonary Disease. TNM: Tumor, Node, Metastasis. ASA: American Society of Anesthesiologists.

**Table 2:** Immune Cell Profiles by Obstruction Status and T stage.

	T1–3			T4		
Immune Cells	No Obstruction *n* = 127	with Obstruction *n* = 105	*p*-Value ^1^	No Obstruction *n* = 37	with Obstruction *n* = 59	*p*-Value ^1^
WBC (×10^9^/L)			0.569			0.757
Mean ± SD	6.88 ± 3.49	6.37 ± 2.50		6.40 ± 2.76	6.39 ± 2.76	
Neutrophils (×10^9^/L)			0.994			0.433
Mean ± SD	4.87 ± 3.30	4.49 ± 2.55		4.23 ± 3.02	4.48 ± 2.55	
Lymphocytes (×10^9^/L)			0.119			**<0.001**
Mean ± SD	1.37 ± 0.56	1.29 ± 0.59		1.55 ± 0.48	1.22 ± 0.42	
Monocytes (×10^9^/L)			0.873			0.057
Mean ± SD	0.49 ± 0.24	0.46 ± 0.18		0.42 ± 0.10	0.53 ± 0.28	
Basophils (×10^9^/L)			0.395			0.991
Mean ± SD	0.02 ± 0.02	0.03 ± 0.04		0.03 ± 0.01	0.03 ± 0.02	
Eosinophils (×10^9^/L)			**0.005**			0.212
Mean ± SD	0.12 ± 0.09	0.11 ± 0.14		0.17 ± 0.16	0.14 ± 0.14	

^1^ Wilcoxon rank sum test. *p* less than 0.05 were considered statistically significant and are shown in bold. *n*: Sample size. T1–3, T4: Tumor Stage. WBC: White Blood Cell. SD: Standard Deviation.

**Table 3:** Cox Regression Analysis—DFS.

Disease-Free Survival (DFS) Analysis				
	Univariate Analysis		Multivariate Analysis	
Variable	Univariate HR (95% CI)	Univariate *p*	Multivariate HR (95% CI)	Multivariate *p*
Obstruction	1.25 (0.86–1.83)	0.2		
Age	1.02 (1.00–1.04)	**0.016**	1.01 (1.00–1.03)	0.2
Gender	1.02 (0.70–1.49)	>0.9		
Tumor location	-	0.5		
Colon	-	-		
Rectum	0.85 (0.55–1.32)	-		
Differentiation	-	0.8		
High/Other	-	-		
Moderate	1.00 (0.57–1.73)	-		
Low	1.20 (0.60–2.38)	-		
T stage	-	**<0.001**	-	-
I-II	-	-	-	-
III	1.98 (1.01–3.89)	-	2.02 (1.01–4.04)	**0.047**
IV	4.11 (2.06–8.22)	-	3.39 (1.61–7.13)	**0.001**
N stage	-	**0.039**		
N0	-	-		
N1–2	1.50 (1.01–2.21)	-	1.31 (0.81–2.11)	0.3
M stage	-	**0.031**		
M0	-	-		
M1	3.22 (1.31–7.94)	-	1.52 (0.54–4.27)	0.4
Postoperative radiotherapy	0.74 (0.18–3.02)	0.7		
Postoperative chemotherapy	0.85 (0.58–1.24)	0.4		
Surgical approach	-	**0.034**		
Laparoscope	-	-		
Open	1.50 (1.03–2.20)	-	1.08 (0.71–1.64)	0.7
Blood transfusion	1.67 (1.13–2.45)	**0.011**	1.65 (1.08–2.54)	**0.022**
Primary anastomosis	0.42 (0.28–0.63)	**<0.001**	0.53 (0.34–0.84)	**0.007**
Vascular embolus	1.54 (0.98–2.42)	0.069	0.83 (0.47–1.43)	0.5
Nerve invasion	1.77 (1.18–2.66)	**0.008**	1.51 (0.94–2.42)	0.087
Total lymph nodes	0.98 (0.96–1.00)	**0.036**	0.98 (0.96–1.00)	0.082
Positive lymph nodes	1.05 (1.00–1.09)	0.065	1.00 (0.94–1.06)	>0.9
CD4*+* (Invasive margin)	0.79 (0.63–0.99)	**0.029**	0.94 (0.73–1.20)	0.6
CD8^+^ (Invasive margin)	1.04 (0.90–1.20)	0.6		
CD20^+^ (Invasive margin)	1.01 (0.84–1.22)	>0.9		
CD68^+^ (Invasive margin)	0.56 (0.43–0.73)	**<0.001**	0.59 (0.40–0.87)	**0.008**
CD4^+^ (Central tumor)	0.94 (0.73–1.20)	0.6		
CD8^+^ (Central tumor)	1.10 (0.97–1.26)	0.2		
CD20^+^ (Central tumor)	1.11 (0.99–1.24)	0.14		
CD68^+^ (Central tumor)	0.77 (0.62–0.95)	**0.010**	1.13 (0.83–1.54)	0.4

Univariate and multivariate Cox regression analyses. HR = hazard ratio; CI = confidence interval. *p* less than 0.05 were considered statistically significant and are shown in bold.

**Table 4:** Cox Regression Analysis—OS.

Overall Survival (OS) Analysis				
	Univariate Analysis		Multivariate Analysis	
Variable	Univariate HR (95% CI)	Univariate *p*	Multivariate HR (95% CI)	Multivariate *p*
Obstruction	1.60 (1.07–2.40)	**0.021**	1.26 (0.80–1.99)	0.3
Age	1.03 (1.01–1.05)	**0.001**	1.02 (1.00–1.04)	0.090
Gender	0.95 (0.64–1.42)	0.8		
Tumor location	-	0.5		
Colon	-	-		
Rectum	0.86 (0.54–1.37)	-		
Differentiation	-	0.3		
High/Other	-	-		
Moderate	0.83 (0.47–1.46)	-		
Low	1.24 (0.62–2.46)	-		
T stage	-	**<0.001**	-	-
I-II	-	-	-	-
III	2.07 (0.98–4.38)	-	2.01 (0.93–4.33)	0.074
IV	4.34 (2.03–9.28)	-	3.29 (1.46–7.39)	**0.004**
N stage	-	**0.001**	-	-
N0	-	-	-	-
N1–2	1.98 (1.29–3.04)	-	1.93 (1.15–3.25)	**0.013**
M stage	-	0.15		
M0	-	-		
M1	2.32 (0.85–6.32)	-		
Postoperative radiotherapy	0.41 (0.06–2.97)	0.3		
Postoperative chemotherapy	0.66 (0.44–0.99)	**0.043**	0.72 (0.45–1.15)	0.2
Surgical approach	-	**<0.001**	-	-
Laparoscope	-	-	-	-
Open	2.11 (1.39–3.21)	-	1.55 (0.98–2.44)	0.058
Blood transfusion	1.99 (1.33–2.98)	**0.001**	1.92 (1.22–3.02)	**0.005**
Primary anastomosis	0.34 (0.22–0.51)	**<0.001**	0.54 (0.34–0.86)	**0.010**
Vascular embolus	1.64 (1.03–2.63)	**0.048**	1.03 (0.57–1.84)	>0.9
Nerve invasion	1.59 (1.03–2.45)	**0.043**	1.29 (0.78–2.16)	0.3
Total lymph nodes	0.97 (0.95–1.00)	**0.011**	0.97 (0.94–1.00)	**0.024**
Positive lymph nodes	1.07 (1.02–1.12)	**0.007**	1.01 (0.95–1.07)	0.8
CD4^+^ (Invasive margin)	0.81 (0.64–1.02)	0.064	1.04 (0.78–1.38)	0.8
CD8^+^ (Invasive margin)	1.04 (0.90–1.21)	0.6		
CD20^+^ (Invasive margin)	0.98 (0.78–1.22)	0.8		
CD68^+^ (Invasive margin)	0.49 (0.36–0.66)	**<0.001**	0.60 (0.39–0.94)	**0.024**
CD4^+^ (Central tumor)	0.97 (0.76–1.24)	0.8		
CD8^+^ (Central tumor)	0.99 (0.81–1.20)	>0.9		
CD20^+^ (Central tumor)	1.13 (1.01–1.27)	0.077	1.02 (0.89–1.16)	0.8
CD68^+^ (Central tumor)	0.66 (0.51–0.84)	**<0.001**	0.93 (0.66–1.31)	0.7

Univariate and multivariate Cox regression analyses. *p* less than 0.05 were considered statistically significant and are shown in bold. HR = hazard ratio; CI = confidence interval.

**Table 5:** Cox Regression Analysis—DFS.

Disease-Free Survival (DFS) Analysis				
	Univariate Analysis		Multivariate Analysis	
Variable	Univariate HR (95% CI)	Univariate *p*	Multivariate HR (95% CI)	Multivariate *p*
Age	1.02 (1.00–1.05)	0.09	1.04 (1.01–1.07)	0.013
Gender	1.00 (0.57–1.73)	>0.9		
Tumor location	-	0.8		
Colon	-	-		
Rectum	0.93 (0.50–1.75)	-		
Differentiation	-	0.6		
High/Other	-	-		
Moderate	1.16 (0.49–2.75)	-		
Low	1.64 (0.58–4.61)	-		
T stage	-	**<0.001**	-	-
I–II	-	-	-	-
III	5.65 (1.35–23.6)	-	6.59 (1.51–28.7)	**0.012**
IV	10.2 (2.35–44.3)	-	10.1 (2.21–46.0)	**0.003**
N stage	-	**0.027**	-	-
N0	-	-	-	-
N1–2	1.91 (1.06–3.46)	-	1.78 (0.83–3.81)	0.14
M stage	-	0.082	-	-
M0	-	-	-	-
M1	4.78 (1.14–20.0)	-	6.70 (1.39–32.2)	**0.018**
Postoperative radiotherapy	0.88 (0.12–6.42)	>0.9		
Postoperative chemotherapy	0.88 (0.51–1.53)	0.6		
Surgical approach	-	**0.025**	-	-
Laparoscope	-	-	-	-
Open	1.91 (1.07–3.42)	-	1.36 (0.73–2.55)	0.3
Blood transfusion	1.43 (0.82–2.51)	0.2		
Primary anastomosis	0.46 (0.25–0.86)	**0.021**	0.50 (0.24–1.05)	0.068
Vascular embolus	1.63 (0.83–3.19)	0.2		
Nerve invasion	2.01 (1.09–3.71)	**0.034**	1.51 (0.77–2.96)	0.2
Total lymph nodes	0.98 (0.96–1.01)	0.3		
Positive lymph nodes	1.13 (1.05–1.21)	**0.003**	1.07 (0.97–1.18)	0.2
CD4^+^ (Invasive margin)	0.62 (0.41–0.93)	**0.008**	1.18 (0.58–2.43)	0.6
CD8^+^ (Invasive margin)	1.05 (0.86–1.29)	0.6		
CD20^+^ (Invasive margin)	0.64 (0.46–0.89)	**0.005**	0.95 (0.65–1.41)	0.8
CD68^+^ (Invasive margin)	0.53 (0.36–0.79)	**<0.001**	0.68 (0.40–1.16)	0.2
CD4^+^ (Central tumor)	0.69 (0.48–1.01)	**0.033**	0.75 (0.43–1.32)	0.3
CD8^+^ (Central tumor)	1.15 (0.95–1.39)	0.2		
CD20^+^ (Central tumor)	0.84 (0.62–1.15)	0.3		
CD68^+^ (Central tumor)	0.58 (0.39–0.84)	**0.001**	0.79 (0.48–1.31)	0.4

Univariate and multivariate Cox regression analyses. HR = hazard ratio; CI = confidence interval. *p* less than 0.05 were considered statistically significant and are shown in bold.

**Table 6:** Cox Regression Analysis—OS.

Overall Survival (OS) Analysis				
	Univariate Analysis		Multivariate Analysis	
Variable	Univariate HR (95% CI)	Univariate *p*	Multivariate HR (95% CI)	Multivariate *p*
Age	1.04 (1.01–1.07)	**0.015**	1.05 (1.01–1.09)	0.017
Gender	0.84 (0.45–1.57)	0.6		
Tumor location	-	>0.9		
Colon	-	-		
Rectum	0.97 (0.49–1.95)	-		
Differentiation	-	0.3		
High/Other	-	-		
Moderate	0.78 (0.32–1.89)	-		
Low	1.52 (0.54–4.29)	-		
T stage	-	**0.003**	-	-
I–II	-	-	-	-
III	4.21 (1.00–17.8)	-	4.60 (1.04–20.3)	**0.044**
IV	7.95 (1.80–35.1)	-	7.24 (1.54–34.1)	**0.012**
N stage	-	**0.010**	-	-
N0	-	-	-	-
N1–2	2.38 (1.19–4.75)	-	2.53 (1.03–6.23)	**0.044**
M stage	-	0.6		
M0	-	-		
M1	1.95 (0.27–14.3)	-		
Postoperative radiotherapy	0 (0.00-Inf)	0.2		
Postoperative chemotherapy	0.52 (0.27–1.00)	**0.044**	0.54 (0.26–1.12)	0.10
Surgical approach	-	**0.004**	-	-
Laparoscope	-	-	-	-
Open	2.62 (1.31–5.22)	-	2.05 (0.95–4.41)	0.066
Blood transfusion	2.28 (1.24–4.21)	**0.010**	1.27 (0.64–2.52)	0.5
Primary anastomosis	0.29 (0.16–0.56)	**<0.001**	0.50 (0.22–1.12)	0.092
Vascular embolus	1.44 (0.66–3.12)	0.4		
Nerve invasion	1.47 (0.71–3.01)	0.3		
Total lymph nodes	0.98 (0.95–1.02)	0.3		
Positive lymph nodes	1.17 (1.09–1.25)	**<0.001**	1.11 (0.99–1.25)	0.066
CD4^+^ (Invasive margin)	0.67 (0.44–1.03)	**0.041**	1.13 (0.71–1.81)	0.6
CD8^+^ (Invasive margin)	1.1 (0.92–1.32)	0.4		
CD20^+^ (Invasive margin)	0.5 (0.33–0.76)	**<0.001**	0.82 (0.52–1.30)	0.4
CD68^+^ (Invasive margin)	0.51 (0.32–0.80)	**<0.001**	0.73 (0.41–1.29)	0.3
CD4^+^ (Central tumor)	0.83 (0.58–1.19)	0.3		
CD8^+^ (Central tumor)	0.97 (0.68–1.37)	0.9		
CD20^+^ (Central tumor)	0.91 (0.66–1.27)	0.6		
CD68^+^ (Central tumor)	0.59 (0.39–0.90)	**0.005**	0.70 (0.38–1.27)	0.2

Univariate and multivariate Cox regression analyses. HR = hazard ratio; CI = confidence interval. *p* less than 0.05 were considered statistically significant and are shown in bold.

## Data Availability

The original contributions presented in this study are included in the article/Supplementary Material. Further inquiries can be directed to the corresponding authors.

## Supplementary Materials

The following supporting information can be downloaded at: https://www.mdpi.com/article/10.3390/biomedicines13112596/s1, Table S1: Immune Cell Profiles by Obstruction Status and Age; Table S2: Immune Cell Profiles by Obstruction Status and Sex; Table S3: Immune Cell Profiles by Obstruction Status and N Stage; Table S4: Immune Cell Profiles by Obstruction Status and TNM Stage; Table S5: Immune Cell Profiles by Obstruction Status and Tumor Location; Table S6: Immune Cell Profiles by Obstruction Status and Differentiation; Table S7: Immune Cell Profiles by Obstruction Status and Nerve Invasion; Table S8: Immune Cell Profiles by Obstruction Status and Vascular Embolus; Table S9: Immune Cell Profiles by Obstruction Status and Tumor Size.

## References

[1] Brenner H., Kloor M., Pox C.P. (2014). Colorectal Cancer. Lancet.

[2] Weng J., Li S., Zhu Z., Liu Q., Zhang R., Yang Y., Li X. (2022). Exploring Immunotherapy in Colorectal Cancer. J. Hematol. Oncol..

[3] Mariani F. (2014). Inflammatory Pathways in the Early Steps of Colorectal Cancer Development. World J. Gastroenterol..

[4] Deng S., Wang J., Zou F., Cheng D., Chen M., Gu J., Shi J., Yang J., Xue Y., Jiang Z. (2025). Palmitic Acid Accumulation Activates Fibroblasts and Promotes Matrix Stiffness in Colorectal Cancer. Cancer Res..

[5] Zhang X., Yu D., Wu D., Gao X., Shao F., Zhao M., Wang J., Ma J., Wang W., Qin X. (2023). Tissue-Resident Lachnospiraceae Family Bacteria Protect against Colorectal Carcinogenesis by Promoting Tumor Immune Surveillance. Cell Host Microbe.

[6] El Sissy C., Kirilovsky A., Lagorce Pagès C., Marliot F., Custers P.A., Dizdarevic E., Sroussi M., Castillo-Martin M., Haicheur N., Dermani M. (2024). International Validation of the Immunoscore Biopsy in Patients with Rectal Cancer Managed by a Watch-and-Wait Strategy. J. Clin. Oncol..

[7] Angell H.K., Bruni D., Barrett J.C., Herbst R., Galon J. (2020). The Immunoscore: Colon Cancer and Beyond. Clin. Cancer Res..

[8] Dienstmann R., Vermeulen L., Guinney J., Kopetz S., Tejpar S., Tabernero J. (2017). Consensus Molecular Subtypes and the Evolution of Precision Medicine in Colorectal Cancer. Nat. Rev. Cancer.

[9] Guinney J., Dienstmann R., Wang X., De Reyniès A., Schlicker A., Soneson C., Marisa L., Roepman P., Nyamundanda G., Angelino P. (2015). The Consensus Molecular Subtypes of Colorectal Cancer. Nat. Med..

[10] Overman M.J., Gelsomino F., Aglietta M., Wong M., Limon Miron M.L., Leonard G., García-Alfonso P., Hill A.G., Cubillo Gracian A., Van Cutsem E. (2024). Nivolumab plus Relatlimab in Patients with Previously Treated Microsatellite Instability-High/Mismatch Repair-Deficient Metastatic Colorectal Cancer: The Phase II CheckMate 142 Study. J. Immunother. Cancer.

[11] Bankhead P., Loughrey M.B., Fernández J.A., Dombrowski Y., McArt D.G., Dunne P.D., McQuaid S., Gray R.T., Murray L.J., Coleman H.G. (2017). QuPath: Open Source Software for Digital Pathology Image Analysis. Sci. Rep..

[12] Liu W., Kuang T., Liu L., Deng W. (2024). The Role of Innate Immune Cells in the Colorectal Cancer Tumor Microenvironment and Advances in Anti-Tumor Therapy Research. Front. Immunol..

[13] Melero I., Rouzaut A., Motz G.T., Coukos G. (2014). T-Cell and NK-Cell Infiltration into Solid Tumors: A Key Limiting Factor for Efficacious Cancer Immunotherapy. Cancer Discov..

[14] Binnewies M., Roberts E.W., Kersten K., Chan V., Fearon D.F., Merad M., Coussens L.M., Gabrilovich D.I., Ostrand-Rosenberg S., Hedrick C.C. (2018). Understanding the Tumor Immune Microenvironment (TIME) for Effective Therapy. Nat. Med..

[15] Lamplugh Z., Fan Y. (2021). Vascular Microenvironment, Tumor Immunity and Immunotherapy. Front. Immunol..

[16] Dai Q., Wu W., Amei A., Yan X., Lu L., Wang Z. (2021). Regulation and Characterization of Tumor-Infiltrating Immune Cells in Breast Cancer. Int. Immunopharmacol..

[17] Decock J., Comito G., Zaravinos A. (2023). Editorial: Tumor Microenvironment, Inflammation, and Resistance to Immunotherapies. Front. Oncol..

[18] Mezheyeuski A., Micke P., Martín-Bernabé A., Backman M., Hrynchyk I., Hammarström K., Ström S., Ekström J., Edqvist P.-H., Sundström M. (2021). The Immune Landscape of Colorectal Cancer. Cancers.

[19] Zhou X., Wang G., Tian C., Du L., Prochownik E.V., Li Y. (2024). Inhibition of DUSP18 Impairs Cholesterol Biosynthesis and Promotes Anti-Tumor Immunity in Colorectal Cancer. Nat. Commun..

[20] Yoon P.S., Del Piccolo N., Shirure V.S., Peng Y., Kirane A., Canter R.J., Fields R.C., George S.C., Gholami S. (2021). Advances in Modeling the Immune Microenvironment of Colorectal Cancer. Front. Immunol..

[21] Xu X., Ma J., Yu G., Qiu Q., Zhang W., Cao F. (2021). Effective Predictor of Colorectal Cancer Survival Based on Exclusive Expression Pattern Among Different Immune Cell Infiltration. J. Histochem. Cytochem..

[22] Subtil B., Cambi A., Tauriello D.V.F., De Vries I.J.M. (2021). The Therapeutic Potential of Tackling Tumor-Induced Dendritic Cell Dysfunction in Colorectal Cancer. Front. Immunol..

[23] Tuomisto A.E., Mäkinen M.J., Väyrynen J.P. (2019). Systemic Inflammation in Colorectal Cancer: Underlying Factors, Effects, and Prognostic Significance. World J. Gastroenterol..

[24] De La Cruz-Merino L., Henao Carrasco F., Vicente Baz D., Nogales Fernández E., Reina Zoilo J.J., Codes Manuel De Villena M., Pulido E.G. (2011). Immune Microenvironment in Colorectal Cancer: A New Hallmark to Change Old Paradigms. Clin. Dev. Immunol..

[25] Haq M.F., Bhat G.A., Wani M.A., Malik A.A., Ul Haq M.I., Ul Haq M.E. (2024). Outcome of Obstructing vs Nonobstructing Colorectal Carcinomas: Comparative Study at Tertiary Care Hospital in Kashmir. Euroasian J. Hepato-Gastroenterol..

[26] Zhang L., Li Z., Skrzypczynska K.M., Fang Q., Zhang W., O’Brien S.A., He Y., Wang L., Zhang Q., Kim A. (2020). Single-Cell Analyses Inform Mechanisms of Myeloid-Targeted Therapies in Colon Cancer. Cell.

[27] Cario E., Gerken G., Podolsky D.K. (2007). Toll-Like Receptor 2 Controls Mucosal Inflammation by Regulating Epithelial Barrier Function. Gastroenterology.

