# Supplementary material for: Analysis of Immune Cell Infiltration Distribution and Prognostic Value in Obstructive Colorectal Cancer
Source: Biomedicines. 2025 Oct 23;13(11):2596. doi: 10.3390/biomedicines13112596 (PMC12650382; doi:10.3390/biomedicines13112596)
Supplement: Supplementary file 1 [file biomedicines-13-02596-s001.zip › Table_S1-S9.pdf]

Table S1. Immune Cell Profiles by Obstruction Status and Age

	<60			≥60		
**Immune Cells**	**No Obstruction** N = 57	**With Obstruction** N = 60	**p-value**[1]	**No Obstruction** N = 107	**With Obstruction** N = 104	**p-value**[1]
**WBC (×10⁹/L)**			0.182			0.066
Mean ± SD	6.96 ± 3.59	7.08 ± 2.70		6.67 ± 3.20	5.97 ± 2.45	
**Neutrophils (×10⁹/L)**			0.103			0.252
Mean ± SD	4.98 ± 3.80	5.21 ± 2.86		4.60 ± 2.92	4.06 ± 2.25	
**Lymphocytes (×10⁹/L)**			0.065			0.027
Mean ± SD	1.37 ± 0.54	1.26 ± 0.61		1.43 ± 0.56	1.26 ± 0.49	
**Monocytes (×10⁹/L)**			0.580			0.484
Mean ± SD	0.47 ± 0.21	0.46 ± 0.18		0.48 ± 0.23	0.50 ± 0.25	
**Basophils (×10⁹/L)**			0.937			0.638
Mean ± SD	0.02 ± 0.01	0.03 ± 0.02		0.03 ± 0.02	0.03 ± 0.04	
**Eosinophils (×10⁹/L)**			0.084			0.026
Mean ± SD	0.11 ± 0.10	0.11 ± 0.16		0.14 ± 0.12	0.12 ± 0.13	

[1]Wilcoxon rank sum test

Table S2. Immune Cell Profiles by Obstruction Status and Sex

	Male			Female		
**Immune Cells**	**No Obstruction** N = 100	**With Obstruction** N = 85	**p-value**[1]	**No Obstruction** N = 64	**With Obstruction** N = 79	**p-value**[1]
**WBC (×10^9/L)**			0.328			0.062
Mean ± SD	6.70 ± 3.37	6.81 ± 2.83		6.89 ± 3.30	5.91 ± 2.24	
**Neutrophils (×10^9/L)**			0.087			0.137
Mean ± SD	4.65 ± 3.16	4.93 ± 2.69		4.85 ± 3.39	4.00 ± 2.30	
**Lymphocytes (×10^9/L)**			0.001			0.329
Mean ± SD	1.41 ± 0.56	1.16 ± 0.46		1.41 ± 0.53	1.37 ± 0.59	
**Monocytes (×10^9/L)**			0.003			0.039
Mean ± SD	0.48 ± 0.25	0.55 ± 0.26		0.47 ± 0.17	0.41 ± 0.14	
**Basophils (×10^9/L)**			0.874			0.245
Mean ± SD	0.02 ± 0.01	0.03 ± 0.03		0.03 ± 0.02	0.03 ± 0.04	
**Eosinophils (×10^9/L)**			0.041			0.022
Mean ± SD	0.13 ± 0.13	0.13 ± 0.17		0.12 ± 0.08	0.10 ± 0.10	

[1]Wilcoxon rank sum test

Table S3. Immune Cell Profiles by Obstruction Status and N Stage

	N0			N1-2		
**Immune Cells**	**No Obstruction** N = 63	**With Obstruction** N = 83	**p-value**[1]	**No Obstruction** N = 101	**With Obstruction** N = 81	**p-value**[1]
**WBC (×10⁹/L)**			0.227			0.548
Mean ± SD	6.77 ± 2.94	6.46 ± 2.82		6.77 ± 3.57	6.29 ± 2.35	
**Neutrophils (×10⁹/L)**			0.442			0.206
Mean ± SD	4.74 ± 2.99	4.55 ± 2.70		4.72 ± 3.40	4.42 ± 2.38	
**Lymphocytes (×10⁹/L)**			0.116			0.015
Mean ± SD	1.41 ± 0.59	1.26 ± 0.45		1.42 ± 0.53	1.27 ± 0.61	
**Monocytes (×10⁹/L)**			0.489			0.628
Mean ± SD	0.45 ± 0.17	0.50 ± 0.27		0.49 ± 0.25	0.46 ± 0.17	
**Basophils (×10⁹/L)**			0.733			0.790
Mean ± SD	0.03 ± 0.02	0.04 ± 0.04		0.02 ± 0.01	0.03 ± 0.02	
**Eosinophils (×10⁹/L)**			0.003			0.267
Mean ± SD	0.14 ± 0.14	0.12 ± 0.15		0.12 ± 0.09	0.12 ± 0.13	

[1]Wilcoxon rank sum test

Table S4. Immune Cell Profiles by Obstruction Status and TNM Stage

	I-II			III-IV		
**Immune Cells**	**No Obstruction** N = 58	**With Obstruction** N = 81	**p-value**[1]	**No Obstruction** N = 106	**With Obstruction** N = 83	**p-value**[1]
**WBC (×10^9/L)**			0.328			0.711
Mean ± SD	6.82 ± 3.05	6.50 ± 2.85		6.74 ± 3.49	6.26 ± 2.33	
**Neutrophils (×10^9/L)**			0.579			0.327
Mean ± SD	4.80 ± 3.11	4.60 ± 2.72		4.69 ± 3.33	4.37 ± 2.37	
**Lymphocytes (×10^9/L)**			0.128			0.017
Mean ± SD	1.40 ± 0.61	1.24 ± 0.45		1.42 ± 0.51	1.28 ± 0.61	
**Monocytes (×10^9/L)**			0.577			0.502
Mean ± SD	0.45 ± 0.17	0.50 ± 0.27		0.48 ± 0.24	0.47 ± 0.17	
**Basophils (×10^9/L)**			0.666			0.900
Mean ± SD	0.03 ± 0.02	0.04 ± 0.04		0.02 ± 0.01	0.03 ± 0.02	
**Eosinophils (×10^9/L)**			0.006			0.196
Mean ± SD	0.14 ± 0.15	0.12 ± 0.15		0.12 ± 0.09	0.12 ± 0.13	

[1]Wilcoxon rank sum test

Table S5. Immune Cell Profiles by Obstruction Status and Tumor Location

	Colon			Rectum		
**Immune Cells**	**No Obstruction** N = 111	**With Obstruction** N = 124	**p-value**[1]	**No Obstruction** N = 53	**With Obstruction** N = 40	**p-value**[1]
**WBC (×10^9/L)**			0.827			0.210
Mean ± SD	6.61 ± 3.23	6.45 ± 2.54		7.11 ± 3.55	6.16 ± 2.76	
**Neutrophils (×10^9/L)**			0.121			0.066
Mean ± SD	4.57 ± 3.25	4.60 ± 2.37		5.07 ± 3.24	4.12 ± 3.03	
**Lymphocytes (×10^9/L)**			<0.001			0.991
Mean ± SD	1.43 ± 0.55	1.21 ± 0.48		1.38 ± 0.55	1.42 ± 0.67	
**Monocytes (×10^9/L)**			0.144			0.426
Mean ± SD	0.46 ± 0.20	0.50 ± 0.23		0.50 ± 0.26	0.45 ± 0.20	
**Basophils (×10^9/L)**			0.780			0.488
Mean ± SD	0.03 ± 0.01	0.03 ± 0.04		0.02 ± 0.02	0.02 ± 0.01	
**Eosinophils (×10^9/L)**			<0.001			0.139
Mean ± SD	0.13 ± 0.12	0.11 ± 0.15		0.12 ± 0.11	0.15 ± 0.11	

[1]Wilcoxon rank sum test

Table S6. Immune Cell Profiles by Obstruction Status and Differentiation

	High			Moderate			Low/Other		
**Immune Cells**	**No Obstruction** N = 18	**With Obstruction** N = 26	**p-value**[1]	**No Obstruction** N = 123	**With Obstruction** N = 118	**p-value**[1]	**No Obstruction** N = 23	**With Obstruction** N = 20	**p-value**[1]
**WBC (×10⁹/L)**			0.148			0.971			0.534
Mean ± SD	6.98 ± 4.06	5.13 ± 1.41		6.76 ± 3.35	6.56 ± 2.63		6.69 ± 2.72	6.95 ± 3.12	
**Neutrophils (×10⁹/L)**			0.246			0.590			0.247
Mean ± SD	5.11 ± 3.86	3.30 ± 1.05		4.71 ± 3.23	4.67 ± 2.68		4.54 ± 2.91	4.92 ± 2.73	
**Lymphocytes (×10⁹/L)**			0.591			0.003			0.257
Mean ± SD	1.20 ± 0.59	1.24 ± 0.47		1.42 ± 0.55	1.24 ± 0.57		1.52 ± 0.47	1.40 ± 0.38	
**Monocytes (×10⁹/L)**			0.565			0.277			0.884
Mean ± SD	0.48 ± 0.16	0.44 ± 0.16		0.47 ± 0.23	0.50 ± 0.24		0.48 ± 0.23	0.47 ± 0.19	
**Basophils (×10⁹/L)**			0.253			0.075			0.085
Mean ± SD	0.03 ± 0.02	0.03 ± 0.02		0.03 ± 0.02	0.03 ± 0.03		0.02 ± 0.01	0.05 ± 0.06	
**Eosinophils (×10⁹/L)**			0.260			0.009			0.218
Mean ± SD	0.16 ± 0.12	0.12 ± 0.10		0.13 ± 0.12	0.12 ± 0.15		0.12 ± 0.10	0.10 ± 0.15	

[1]Wilcoxon rank sum test

Table S7. Immune Cell Profiles by Obstruction Status and Nerve Invasion

	No			Yes		
**Immune Cells**	**No Obstruction** N = 127	**With Obstruction** N = 117	**p-value**[1]	**No Obstruction** N = 37	**With Obstruction** N = 47	**p-value**[1]
**WBC (×10⁹/L)**			0.878			0.488
Mean ± SD	6.78 ± 3.32	6.39 ± 2.36		6.75 ± 3.43	6.34 ± 3.12	
**Neutrophils (×10⁹/L)**			0.609			0.871
Mean ± SD	4.75 ± 3.16	4.48 ± 2.28		4.66 ± 3.55	4.48 ± 3.13	
**Lymphocytes (×10⁹/L)**			0.010			0.048
Mean ± SD	1.40 ± 0.54	1.25 ± 0.58		1.46 ± 0.57	1.28 ± 0.41	
**Monocytes (×10⁹/L)**			0.415			0.534
Mean ± SD	0.48 ± 0.23	0.50 ± 0.25		0.45 ± 0.20	0.44 ± 0.15	
**Basophils (×10⁹/L)**			0.350			0.012
Mean ± SD	0.02 ± 0.02	0.03 ± 0.03		0.03 ± 0.02	0.03 ± 0.04	
**Eosinophils (×10⁹/L)**			0.028			0.028
Mean ± SD	0.13 ± 0.12	0.12 ± 0.15		0.14 ± 0.10	0.10 ± 0.11	

[1]Wilcoxon rank sum test

Table S8. Immune Cell Profiles by Obstruction Status and Vascular Embolus

	No			Yes		
**Immune Cells**	**No Obstruction** N = 137	**With Obstruction** N = 132	**p-value**[1]	**No Obstruction** N = 27	**With Obstruction** N = 32	**p-value**[1]
**WBC ($\times 10^9$/L)**			0.561			0.867
Mean ± SD	6.87 ± 3.43	6.30 ± 2.34		6.26 ± 2.83	6.71 ± 3.49	
**Neutrophils ($\times 10^9$/L)**			0.879			0.743
Mean ± SD	4.83 ± 3.29	4.42 ± 2.27		4.23 ± 2.99	4.74 ± 3.50	
**Lymphocytes ($\times 10^9$/L)**			0.001			0.951
Mean ± SD	1.41 ± 0.54	1.23 ± 0.56		1.42 ± 0.60	1.38 ± 0.41	
**Monocytes ($\times 10^9$/L)**			0.266			0.692
Mean ± SD	0.48 ± 0.23	0.50 ± 0.24		0.42 ± 0.13	0.41 ± 0.15	
**Basophils ($\times 10^9$/L)**			0.867			0.656
Mean ± SD	0.03 ± 0.02	0.03 ± 0.04		0.02 ± 0.01	0.02 ± 0.02	
**Eosinophils ($\times 10^9$/L)**			0.002			0.709
Mean ± SD	0.12 ± 0.10	0.11 ± 0.14		0.17 ± 0.17	0.15 ± 0.15	

[1]Wilcoxon rank sum test

Table S9. Immune Cell Profiles by Obstruction Status and Tumor Size

	<5cm			≥5cm		
**Immune Cells**	**No Obstruction** N = 112	**With Obstruction** N = 84	**p-value**[1]	**No Obstruction** N = 52	**With Obstruction** N = 80	**p-value**[1]
**WBC ($\times 10^9$/L)**			0.493			0.032
Mean ± SD	6.42 ± 3.25	6.57 ± 2.87		7.54 ± 3.42	6.18 ± 2.26	
**Neutrophils ($\times 10^9$/L)**			0.180			0.074
Mean ± SD	4.49 ± 3.32	4.72 ± 2.87		5.25 ± 3.03	4.24 ± 2.14	
**Lymphocytes ($\times 10^9$/L)**			0.157			0.002
Mean ± SD	1.35 ± 0.53	1.24 ± 0.45		1.55 ± 0.58	1.28 ± 0.62	
**Monocytes ($\times 10^9$/L)**			0.263			0.270
Mean ± SD	0.43 ± 0.20	0.45 ± 0.19		0.56 ± 0.24	0.51 ± 0.25	
**Basophils ($\times 10^9$/L)**			0.190			0.962
Mean ± SD	0.02 ± 0.01	0.03 ± 0.04		0.03 ± 0.02	0.03 ± 0.02	
**Eosinophils ($\times 10^9$/L)**			0.072			0.013
Mean ± SD	0.12 ± 0.10	0.12 ± 0.15		0.15 ± 0.14	0.12 ± 0.14	

[1]Wilcoxon rank sum test
